# Systematics of the *Osteocephalus* *buckleyi* species complex (Anura, Hylidae) from Ecuador and Peru

**DOI:** 10.3897/zookeys.229.3580

**Published:** 2012-10-18

**Authors:** Santiago R. Ron, Pablo J. Venegas, Eduardo Toral, Morley Read, Diego A. Ortiz, Andrea L. Manzano

**Affiliations:** 1 Museo de Zoología, Escuela de Biología, Pontificia Universidad Católica del Ecuador, Av. 12 de Octubre y Roca, Aptdo. 17-01-2184, Quito, Ecuador; 2 División de Herpetología-Centro de Ornitología y Biodiversidad (CORBIDI), Santa Rita N˚105 Of. 202, Urb. Huertos de San Antonio, Surco, Lima, Perú; 3 Current address: Facultad de Ciencias Ambientales, Universidad Internacional SEK, Quito, Ecuador; 4 Current address: Biology Department, HH227, San Francisco State University, 1600 Holloway Avenue, San Francisco, CA 94132, USA

**Keywords:** Advertisement calls, Amazon, Anura, Cryptic species, Morphology, *Osteocephalus buckleyi*, Phylogeny

## Abstract

We present a new phylogeny, based on DNA sequences of mitochondrial and nuclear genes, for frogs of the genus *Osteocephalus* with emphasis in the *Osteocephalus buckleyi* species complex. Genetic, morphologic, and advertisement call data are combined to define species boundaries and describe new species. The phylogeny shows strong support for: (1) a basal position of *Osteocephalus taurinus* + *Osteocephalus oophagus*, (2) a clade containing phytotelmata breeding species, and (3) a clade that corresponds to the *Osteocephalus buckleyi* species complex. Our results document a large proportion of hidden diversity within a set of populations that were previously treated as a single, widely distributed species, *Osteocephalus buckleyi*. Individuals assignable to *Osteocephalus buckleyi* formed a paraphyletic group relative to *Osteocephalus verruciger* and *Osteocephalus cabrerai* and contained four species, one of which is *Osteocephalus buckleyi* *sensu stricto* and three are new. Two of the new species are shared between Ecuador and Peru (*Osteocephalus vilmae* **sp. n.** and *Osteocephalus cannatellai* **sp. n.**) and one is distributed in the Amazon region of southern Peru (*Osteocephalus germani* **sp. n.**) We discuss the difficulties of using morphological characters to define species boundaries and propose a hypothesis to explain them.

## Abstract (sp)

Presentamos una nueva filogenia, basada en secuencias de ADN de genes nucleares y mitocondriales, para ranas del género *Osteocephalus* con énfasis en el complejo de especies *Osteocephalus buckleyi*. Datos genéticos, morfológicos, y de cantos de anuncio se combinan para definir límites de especies y describir nuevas especies. La filogenia muestra un soporte fuerte para: (1) una posición basal de *Osteocephalus taurinus* + *Osteocephalus oophagus*, (2) un clado que contiene especies con reproducción en fitotelmatas, y (3) un clado que corresponde el complejo de especies *Osteocephalus buckleyi*. Nuestros resultados documentan una gran proporción de diversidad escondida dentro de un grupo de poblaciones que previamente habían sido tratadas como una sola especie ampliamente distribuida, *Osteocephalus buckleyi*. Los individuos asignables a *Osteocephalus buckleyi* formaron un grupo parafilético en relación a *Osteocephalus verruciger* y *Osteocephalus cabrerai* y contuvieron cuatro especies, una de las cuales es *Osteocephalus buckleyi* *sensu scricto* y tres son nuevas. Dos de las nuevas especies están compartidas entre Ecuador y Perú (*Osteocephalus vilmae* **sp. n.** y *Osteocephalus cannatellai* **sp. n.**) y una está en el sur de Perú (*Osteocephalus germani* **sp. n.**) Discutimos las dificultades de usar caracteres morfológicos para definir límites de especies y proponemos una hipótesis para explicarla.

## Introduction

The Upper Amazon region has the highest alpha diversity of amphibians in the World with several sites exceeding 100 species in less than 10 km^2^ (Bass et al. 2010). Remarkably, these figures may vastly underestimate the total diversity as shown by the discovery of large numbers of cryptic species with the use of genetic markers (e.g., Fouquet et al. 2007; Funk et al. 2011; Padial and De la Riva 2009; Ron et al. 2006). These preliminary efforts suggest that the use of genetic characters is crucial to attain a complete understanding of the diversity and evolutionary history of Amazonian amphibians. This necessity is particularly pressing in widespread taxa with pervasive taxonomic problems.

One such group is *Osteocephalus*, a genus of hylid frogs widely distributed in the Amazon Basin, Guianas and upper drainages of the Magdalena and Orinoco rivers (Frost 2010). *Osteocephalus* are arboreal and nocturnal frogs with reproduction modes varying from deposition of eggs in lentic water and exotrophic tadpoles to deposition of eggs in bromeliads and oophagus tadpoles and biparental care (Crump 1974; Jungfer and Weygoldt 1999). There are 24 described species and reports of undescribed species are frequent (e.g., Jungfer 2010; Moravec et al. 2009; Ron et al. 2010). There is only one formally defined species group within *Osteocephalus*, the *Osteocephalus buckleyi* complex. It was first proposed by Cochran and Goin (1970) to allocate *Osteocephalus buckleyi* (Boulenger 1882), *Osteocephalus pearsoni* (Gaige 1929), and *Osteocephalus cabrerai* (Cochran and Goin 1970). Its first large scale review was carried out by Trueb and Duellman (1971) who examined the morphology of specimens from seven countries and concluded that the *Osteocephalus buckleyi* complex (excluding *Osteocephalus verruciger* Werner 1901) consisted of a single, morphologically variable and widely distributed species. They synonymized *Osteocephalus cabrerai*, *Osteocephalus carri* (Cochran and Goin 1970),and *Osteocephalus festae* (Peracca 1904) under *Osteocephalus buckleyi*. The three species have been subsequently resurrected (Duellman and Mendelson 1995; Jungfer 2010; Lynch 2006). Recent reviews (Jungfer 2010; 2011; Moravec et al. 2009; Ron et al. 2010) imply that the *Osteocephalus buckleyi* species complex consists of nine species: *Osteocephalus buckleyi*, *Osteocephalus cabrerai*, *Osteocephalus carri*, *Osteocephalus duellmani* Jungfer 2011, *Osteocephalus festae*, *Osteocephalus inframaculatus* (Boulenger 1882), *Osteocephalus mutabor* Jungfer and Hödl 2002, *Osteocephalus verruciger* and an undescribed species sister to *Osteocephalus verruciger*. A phylogeny based on mitochondrial DNA revealed strong support for the *Osteocephalus buckleyi* complex as well as paraphyly in *Osteocephalus verruciger* and *Osteocephalus buckleyi* (Ron et al. 2010).

Despite recent contributions to the taxonomy of the group (e.g., Jungfer 2010; 2011) the *Osteocephalus buckleyi* species complex still contains undescribed species as well as alpha taxonomic problems (Jungfer 2010; Ron et al. 2010) which attest the difficulties of correctly identifying species boundaries on the basis of morphological evidence alone. Herein we integrate genetic, morphological and advertisement call data to assess the phylogenetic relationships and species boundaries among populations of the *Osteocephalus buckleyi* complex from Ecuador and Peru. The results demonstrate the existence of three new species, which are formally described here.

## Methods

For ease of comparison, we generally follow the format of Trueb and Duellman (1971) for diagnosis and description. Morphological terminology and abbreviations follow Lynch and Duellman (1997). Notation for hand and foot webbing is based on Myers and Duellman (1982). Sex was determined by the texture of dorsal skin, the presence of nuptial pads or vocal sac folds, and by gonadal inspection. Specimens were fixed in 10% formalin and preserved in 70% ethanol. Snout-vent length is abbreviated as SVL. Examined specimens (listed in the type-series and Appendix I) are housed at the collection of the División de Herpetología, Centro de Ornitología y Biodiversidad (CORBIDI), Herpetology Collection at Escuela Politécnica Nacional (EPN-H), Museo de Historia Natural at Universidad San Marcos (MUSM), Museo de Zoología at Pontificia Universidad Católica del Ecuador (QCAZ), and Natural History Museum (BMNH). The pencil drawing of the holotype of *Osteocephalus cannatella* sp. n. was made using a Wild Heerbrugg M3B 10×/21 stereo microscope equipped with a camera lucida.

Principal Components Analysis (PCA) and Discriminant Function Analysis (DFA) were used to assess the degree of morphometric differentiation between species. Only well preserved specimens (Simmons 2002) were measured for the following eight morphological variables, following Duellman (1970): (1) SVL; (2) head length; (3) head width; (4) tympanum diameter; (5) femur length; (6) tibia length; (7) foot length; and (8) eye diameter. All variables were log-transformed. To remove the effect of co-variation with SVL, the PCA was applied to the residuals from the linear regressions between the seven measured variables and SVL. We applied a multivariate analysis of variance (MANOVA) to tests for morphometric differences between sexes. Because we found significant differences in *Osteocephalus buckleyi*, the PCA and DFA were applied on each sex separately. For the PCA, only components with eigenvalues > 1 were retained. The DFA was applied to the measured variables without size correction because we wanted to assess discriminability among species based on all the variables, including SVL. Sample sizes are: *Osteocephalus buckleyi* 24 males, 3 females; *Osteocephalus cabrerai* 7 males; *Osteocephalus cannatellai* sp. n. 33 males, 3 females; *Osteocephalus festae* 7 males, 18 females; *Osteocephalus germani* sp. n. 2 males, 5 females; *Osteocephalus verruciger* 22 males, 5 females; and *Osteocephalus vilmae* sp. n. 4 males. Both PCA and DFA were conducted in JMP® 8.01 (SAS Institute 2008). Measurements were made using digital calipers (to the nearest 0.01 mm).

Advertisement calls recordings were made with a Sennheiser™ ME-67 directional microphone with digital recorder Olympus™ LS10. Calls were analyzed using software Raven 1.2.1 (Charif et al. 2004) at a sampling frequency of 22.1 kHz and a frequency resolution of 21.5 Hz. Calls consist of two components, the first is a rattle note and the second is a quack note. Measured call variables are: (1) call rate: number of calls per second, (2) dominant frequency: frequency with the most energy, measured along all the call, (3) duration of first component note: time from the beginning to the end of note, (4) duration of second component: time from beginning of first quack to the end of the last, (5) first component interval: time from the end of last note of the first component to the beginning of the first note of the second component, (6) number of pulses: number of pulses in a first component note, (7) pulse rate: number of pulses/duration of first component note, (8) duration of second component note: duration from beginning to end of a single quack, (9) quack rate: number of quacks/duration of second component. If available, several calls or notes were analyzed per individual to calculate an individual average. Original recordings are deposited in the audio archive of the QCAZ and are available through the AmphibiaWebEcuador website (http://zoologia.puce.edu.ec/vertebrados/anfibios/).

### DNA extraction, amplification, and sequencing

Total DNA was extracted from muscle or liver tissue preserved in 95% ethanol or tissue storage buffer using standard phenol–chloroform extraction protocols (Sambrook et al. 1989). Polymerase chain reaction (PCR) was used to amplify the mitochondrial genes 12S rRNA ,16S rRNA, ND1 (with flanking tRNA genes), CO1, and control region. We amplified one DNA fragment for 12S, CO1, and the control region and one or two overlapping fragments for the last ~320 bp of 16S and the adjacent ND1 using primers listed in Goebel et al. (1999) and Moen and Wiens (2009). We also amplified the nuclear gene *POMC* as a single fragment using primers listed by Wiens et al. (2005). PCR amplification was carried under standard protocols. Amplified products were sequenced by the Macrogen Sequencing Team (Macrogen Inc., Seoul, Korea).

### Phylogenetic analyses

We estimated phylogenetic relations between species of *Osteocephalus* based on newly generated sequence data for five mitochondrial (*12S* RNA, *CO1*, *16S*, *ND1*, control region) and one nuclear gene (*POMC*) for a total of up to 4170 bp. To expand the species sampling, we also included sequences from GenBank. All samples are listed in Table 1. For the outgroup, we included one sample of *Trachycephalus jordani* and one of *Trachycephalus typhonius* (based on Faivovich et al. 2005 and Wiens et al. 2010). The completeness of the sequences varied considerably among individuals (specially for samples from GenBank which typically lacked three or more loci). Nevertheless, we included samples with missing data because analyses of both empirical and simulated matrices have shown that taxa with missing sequences can be accurately placed in model-based phylogenetic analyses if the number of characters is large, as in our matrix (for a review see Wiens and Morrill 2011).

Preliminary sequence alignment was done with MAFFT 6.814b software with the L-INS-i algorithm (Katoh et al. 2002). The sequence matrix was imported to Mesquite (version 2.72; Maddison and Maddison 2009) and the ambiguously aligned regions were adjusted manually to produce a parsimonious alignment (i.e., informative sites minimized). In protein coding loci, DNA sequences were translated to amino acids with Mesquite to aid the manual alignment. Phylogenetic trees were obtained using Bayesian inference.

Because our dataset includes several loci, it is unlikely that it fits a single model of nucleotide substitution. Thus, we partitioned the data to analyze each partition under a separate model. The best model for each partition was chosen with JModelTest version 0.1.1 (Posada 2008) using the Akaike Information Criterion with sample size correction as optimality measure. We also evaluated three different partition strategies: (i) a single partition, (ii) six partitions (one per loci), and (iii) twelve partitions (one for each codon position in protein coding loci plus one for each non protein coding loci). The best partition strategy was chosen by estimating Bayes factors using a threshold of 10 as evidence in favor of the more complex partition (Brandley et al. 2005).

Each Bayesian analysis consisted of two parallel runs of the Metropolis coupled Monte Carlo Markov chain for 5 × 10^6^ generations. Each run had four chains with a temperature of 0.05. The prior for the rate matrix was a uniform dirichlet and all topologies were equally probable a priori. Convergence into a stationary distribution was determined by reaching average standard deviation split frequencies < 0.05 between runs. We also used software Tracer ver. 1.5 (Rambaut and Drummond 2007) to visually inspect convergence and stationarity of the runs. The first 50% of the sampled generations were discarded as burn-in and the remaining were used to estimate the Bayesian tree, posterior probabilities and other model parameters. Phylogenetic analyses were carried out in MrBayes 3.2.1 (Ronquist et al. 2012).

Because the only nuclear gene analyzed had low variability and few informative sites, it was concatenated to the mitochondrial genes into a single matrix. We recognize the advantages of species-tree methods (e.g., Edwards et al. 2007) but could not use them given the insufficient number of nuclear genes sampled. We encourage the application of those methodologies in future phylogenetic inferences in *Osteocephalus*.

**Table 1.** Genbank accession numbers for DNA sequences used in the phylogenetic analysis.

**Museum No.**	**Species**	**Genbank Accession No.**					**Reference**
		**D*116S-N***	**2*S1***	**O*1C***	**Control Region**	**M*CPO***	
KU 143119	*Osteocephalus alboguttatus*	EU034081	--	--	--	--	Moen and Wiens 2009
QCAZ 15981	*Osteocephalus alboguttatus*	HQ600596	HQ600629	--	JX875680	JX875744	Ron et al. 2010; This study
LAC 2216	*Osteocephalus buckleyi*	EU034082	DQ380378	--	--	EU034116	Moen and Wiens 2009; Wiens et al. 2006
CORBIDI 7458	*Osteocephalus buckleyi*	JX875606	JX847067	JX875806	--	JX875734	This study
CORBIDI 7459	*Osteocephalus buckleyi*	JX875607	JX847068	JX875807	--	JX875735	This study
CORBIDI 7462	*Osteocephalus buckleyi*	JX875608	JX847069	JX875808	JX875657	JX875736	This study
CORBIDI 7516	*Osteocephalus buckleyi*	--	JX847070	--	--	JX875737	This study
LAC 2216	*Osteocephalus buckleyi*	EU034082	DQ380378	--	--	--	Moen and Wiens 2009
QCAZ 14948	*Osteocephalus buckleyi*	JX875611	JX847081	JX875812	JX875718	JX875742	This study
QCAZ 24446	*Osteocephalus buckleyi*	HQ600600	HQ600633	JX875821	JX875708	JX875753	Ron et al. 2010; This study
QCAZ 24447	*Osteocephalus buckleyi*	HQ600601	HQ600634	JX875822	JX875686	JX875754	Ron et al. 2010; This study
QCAZ 28277	*Osteocephalus buckleyi*	HQ600606	HQ600639	JX875831	JX875720	JX875763	Ron et al. 2010; This study
QCAZ 28395	*Osteocephalus buckleyi*	HQ600607	HQ600640	JX875832	JX875677	JX875764	Ron et al. 2010; This study
QCAZ 28427	*Osteocephalus buckleyi*	JX875618	JX847087	JX875833	JX875689	JX875765	This study
QCAZ 36703	*Osteocephalus buckleyi*	JX875625	JX847092	JX875845	JX875722	JX875778	This study
QCAZ 39073	*Osteocephalus buckleyi*	JX875627	JX847094	JX875848	JX875714	JX875782	This study
QCAZ 39074	*Osteocephalus buckleyi*	JX875628	JX847095	JX875849	JX875672	JX875783	This study
QCAZ 39285	*Osteocephalus buckleyi*	JX875629	--	JX875850	JX875694	JX875784	This study
QCAZ 43071	*Osteocephalus buckleyi*	JX875633	JX847099	JX875858	JX875724	JX875793	This study
QCAZ 48093	*Osteocephalus buckleyi*	JX875639	JX847105	JX875864	JX875702	JX875798	This study
QCAZ 48827	*Osteocephalus buckleyi*	JX875640	JX847106	JX875865	JX875703	JX875799	This study
AJC 2566	*Osteocephalus cabrerai*	JX875598	JX847062	JX875801	JX875650	JX875725	This study
AJC 2567	*Osteocephalus cabrerai*	JX875599	JX847063	JX875802	JX875707	JX875726	This study
CORBIDI 120	*Osteocephalus cabrerai*	JX875600	--	--	JX875651	JX875727	This study
CORBIDI 5819	*Osteocephalus cabrerai*	JX875604	JX847066	JX875804	JX875655	JX875731	This study
CORBIDI 5821	*Osteocephalus cabrerai*	JX875605	--	JX875805	JX875656	JX875732	This study
LSUMZ H-13720	*Osteocephalus cabrerai*	--	AY843705	--	--	--	Faivovich et al. 2005
QCAZ 27923	*Osteocephalus cabrerai*	JX875617	JX847086	JX875827	JX875709	JX875760	This study
QCAZ 28231	*Osteocephalus cabrerai*	HQ600621	HQ600654	JX875830	JX875710	JX875762	Ron et al. 2010; This study
CORBIDI 9368	*Osteocephalus cannatellai*	--	JX847072	--	JX875658	--	This study
CORBIDI 9370	*Osteocephalus cannatellai*	JX875643	JX847074	--	JX875660	--	This study
CORBIDI 9394	*Osteocephalus cannatellai*	JX875644	JX847075	--	JX875661	--	This study
CORBIDI 9507	*Osteocephalus cannatellai*	JX875645	JX847077	--	JX875662	--	This study
QCAZ 25469	*Osteocephalus cannatellai*	HQ600617	HQ600650	JX875823	JX875687	JX875755	Ron et al. 2010; This study
QCAZ 31016	*Osteocephalus cannatellai*	JX875621	JX847089	JX875839	JX875712	JX875771	This study
QCAZ 31032	*Osteocephalus cannatellai*	JX875622	JX847090	JX875840	JX875691	JX875772	This study
QCAZ 31033	*Osteocephalus cannatellai*	JX875623	--	JX875841	JX875668	JX875773	This study
QCAZ 32506	*Osteocephalus cannatellai*	HQ600618	HQ600651	JX875843	JX875692	JX875775	Ron et al. 2010; This study
QCAZ 32508	*Osteocephalus cannatellai*	HQ600619	HQ600652	JX875844	JX875693	JX875776	Ron et al. 2010; This study
QCAZ 37175	*Osteocephalus cannatellai*	HQ600620	HQ600653	JX875846	JX875713	JX875779	Ron et al. 2010; This study
QCAZ 39633	*Osteocephalus cannatellai*	JX875630	JX847096	JX875852	JX875678	JX875786	This study
QCAZ 40258	*Osteocephalus cannatellai*	JX875631	JX847097	JX875854	JX875696	JX875788	This study
QCAZ 45909	*Osteocephalus cannatellai*	JX875635	JX847101	JX875860	JX875701	JX875795	This study
QCAZ 46472	*Osteocephalus cannatellai*	JX875638	JX847104	JX875863	JX875717	JX875797	This study
QCAZ 49572	*Osteocephalus cannatellai*	JX875641	JX847107	JX875866	JX875674	JX875800	This study
CBF 6051	*Osteocephalus castaneicola*	--	FJ965300	--	--	--	Moravec et al. 2009
NMP6d 28/2009	*Osteocephalus castaneicola*	--	FJ965302	--	--	--	Moravec et al. 2009
NMP6V 73810/3	*Osteocephalus castaneicola*	--	FJ965301	--	--	--	Moravec et al. 2009
NMP6V 73820	*Osteocephalus castaneicola*	--	FJ965303	--	--	--	Moravec et al. 2009
QCAZ 20711	*Osteocephalus deridens*	JX875613	JX847083	JX875817	JX875699	JX875749	This study
NMP6V 71262/2	*Osteocephalus deridens*	--	FJ965304	--	--	--	Moravec et al. 2009
CORBIDI 623	*Osteocephalus festae*	HQ600616	HQ600649	JX875810	JX875705	JX875733	Ron et al. 2010
CORBIDI 760	*Osteocephalus festae*	--	--	JX875809	--	JX875738	This study
CORBIDI 10461	*Osteocephalus festae*	JX875649	JX847071	--	--	--	This study
CORBIDI 1965	*Osteocephalus festae*	--	JX847064	JX875803	--	JX875728	This study
CORBIDI 9585	*Osteocephalus festae*	JX875647	JX847079	--	--	--	This study
CORBIDI 9587	*Osteocephalus festae*	JX875648	JX847080	--	--	--	This study
QCAZ 38420	*Osteocephalus festae*	HQ600613	HQ600646	JX875847	--	JX875781	Ron et al. 2010; This study
QCAZ 39364	*Osteocephalus festae*	HQ600615	HQ600648	JX875851	JX875715	JX875785	Ron et al. 2010; This study
QCAZ 41039	*Osteocephalus festae*	HQ600614	HQ600647	JX875855	JX875716	JX875790	Ron et al. 2010; This study
QCAZ 20785	*Osteocephalus fuscifacies*	HQ600598	HQ600631	JX875818	JX875685	JX875750	Ron et al. 2010; This study
CORBIDI 5505	*Osteocephalus germani*	JX875603	--	--	JX875654	--	This study
CORBIDI 8267	*Osteocephalus germani*	JX875609	--	--	--	JX875739	This study
CORBIDI 8284	*Osteocephalus germani*	JX875610	--	--	--	JX875740	This study
141 MC	*Osteocephalus leprieurii*	--	EF376031	--	--	--	Salducci et al. 2005
AMNH-A 131254	*Osteocephalus leprieurii*	--	AY843707	--	--	--	Faivovich et al. 2005
CORBIDI 4645	*Osteocephalus mutabor*	JX875601	--	--	JX875652	JX875729	This study
CORBIDI 9369	*Osteocephalus mutabor*	JX875642	JX847073	--	JX875659	--	This study
KU 221930	*Osteocephalus mutabor*	--	DQ380379	--	--	--	Wiens et al. 2006
QCAZ 25603	*Osteocephalus mutabor*	HQ600598	HQ600631	JX875824	JX875676	JX875756	Ron et al. 2010; This study
QCAZ 25684	*Osteocephalus mutabor*	JX875615	JX847085	JX875825	JX875700	JX875757	This study
QCAZ 28223	*Osteocephalus mutabor*	HQ600605	HQ600638	JX875829	JX875682	--	Ron et al. 2010; This study
QCAZ 28646	*Osteocephalus mutabor*	HQ600608	HQ600641	JX875834	JX875721	JX875766	Ron et al. 2010; This study
QCAZ 28647	*Osteocephalus mutabor*	HQ600609	HQ600642	JX875835	JX875675	JX875767	Ron et al. 2010; This study
QCAZ 29430	*Osteocephalus mutabor*	JX875619	JX847088	JX875836	JX875704	JX875768	This study
QCAZ 30925	*Osteocephalus mutabor*	JX875620	--	JX875837	JX875690	JX875769	This study
QCAZ 30926	*Osteocephalus mutabor*	HQ600610	HQ600643	JX875838	JX875711	JX875770	Ron et al. 2010; This study
QCAZ 40253	*Osteocephalus mutabor*	HQ600611	HQ600644	JX875853	JX875695	JX875787	Ron et al. 2010; This study
QCAZ 41030	*Osteocephalus mutabor*	JX875632	JX847098	--	JX875673	JX875789	This study
QCAZ 42999	*Osteocephalus mutabor*	HQ600612	HQ600645	JX875857	JX875723	JX875792	Ron et al. 2010; This study
QCAZ 46470	*Osteocephalus mutabor*	JX875636	JX847102	JX875861	JX875697	--	This study
QCAZ 46471	*Osteocephalus mutabor*	JX875637	JX847103	JX875862	JX875698	JX875796	This study
14 MC	*Osteocephalus oophagus*	--	EF376030	--	--	--	Salducci et al. 2005
MNHN 2001.0828	*Osteocephalus oophagus*	--	AY843708	--	--	--	Faivovich et al. 2005
KU 221933	*Osteocephalus planiceps*	--	DQ380380	--	--	--	Wiens et al. 2006
NMP6V 71174/1	*Osteocephalus planiceps*	--	FJ965305	--	--	--	Moravec et al. 2009
NMP6V 71264/1	*Osteocephalus planiceps*	--	FJ965306	--	--	--	Moravec et al. 2009
NMP6V 71264/2	*Osteocephalus planiceps*	--	FJ965307	--	--	--	Moravec et al. 2009
QCAZ 20797	*Osteocephalus planiceps*	HQ600599	HQ600632	JX875819	JX875665	JX875751	Ron et al. 2010; This study
214 MC	*Osteocephalus taurinus*	--	EF376032	--	--	--	Salducci et al. 2005
JM 2007/60	*Osteocephalus taurinus*	--	FJ965296	--	--	--	Moravec et al. 2009
KU 221941	*Osteocephalus taurinus*	AY819512	AY819380	--	--	--	Wiens et al. 2005
QCAZ 18230	*Osteocephalus taurinus*	HQ600597	HQ600630	JX875815	JX875719	JX875747	Ron et al. 2010; This study
KU 205406	*Osteocephalus taurinus*	--	AY326041	--	--	--	Darst and Cannatella 2004
CORBIDI 9477	*Osteocephalus verruciger*	--	JX847076	--	--	--	This study
CORBIDI 9525	*Osteocephalus verruciger*	JX875646	JX847078	--	--	--	This study
KU 217751	*Osteocephalus verruciger*	--	DQ380381	--	--	--	Wiens et al. 2006
QCAZ 15942	*Osteocephalus verruciger*	HQ600626	HQ600659	JX875813	JX875679	JX875743	Ron et al. 2010; This study
QCAZ 15991	*Osteocephalus verruciger*	HQ600623	HQ600656	JX875814	JX875681	JX875745	Ron et al. 2010; This study
QCAZ 17285	*Osteocephalus verruciger*	JX875612	JX847082	--	JX875706	JX875746	This study
QCAZ 20544	*Osteocephalus verruciger*	HQ600622	HQ600655	JX875816	JX875664	JX875748	Ron et al. 2010; This study
QCAZ 22201	*Osteocephalus verruciger*	JX875614	JX847084	JX875820	JX875666	JX875752	This study
QCAZ 26304	*Osteocephalus verruciger*	JX875616	--	--	--	JX875758	This study
QCAZ 32032	*Osteocephalus verruciger*	HQ600625	HQ600658	JX875842	JX875669	JX875774	Ron et al. 2010; This study
QCAZ 41108	*Osteocephalus verruciger*	HQ600627	HQ600660	JX875856	JX875683	JX875791	Ron et al. 2010; This study
QCAZ 45344	*Osteocephalus verruciger*	JX875634	JX847100	JX875859	JX875684	JX875794	This study
CORBIDI 4773	*Osteocephalus vilmae*	JX875602	JX847065	--	JX875653	JX875730	This study
QCAZ 14947	*Osteocephalus vilmae*	HQ600595	HQ600628	JX875811	JX875663	JX875741	Ron et al. 2010; This study
QCAZ 27816	*Osteocephalus yasuni*	HQ600603	HQ600636	JX875826	JX875688	JX875759	Ron et al. 2010; This study
QCAZ 27998	*Osteocephalus yasuni*	HQ600604	HQ600637	JX875828	JX875667	JX875761	Ron et al. 2010; This study
NMP6d 41/2009	*Osteocephalus* sp.	--	FJ965297	--	--	--	Moravec et al. 2009
NMP6V 72173/1	*Osteocephalus* sp.	--	FJ965299	--	--	--	Moravec et al. 2009
NMP6V 72173/3	*Osteocephalus* sp.	--	FJ965308	--	--	--	Moravec et al. 2009
NMP6V 73105	*Osteocephalus* sp.	--	FJ965298	--	--	--	Moravec et al. 2009
QCAZ 35405	*Trachycephalus jordani*	JX875624	JX847091	--	JX875670	JX875777	This study
QCAZ 38075	*Trachycephalus typhonius*	JX875626	JX847093	--	JX875671	JX875780	This study

## Results

### Phylogenetic analyses

Throughout this section, genetic distances are uncorrected *p*-distances for gene *12S*. The complete data set consists of up to six gene fragments (956 bp of *12S*, 325 bp of *16S*, 693 pb of *CO1*, 579 bp of control region, 1079 bp of *ND1*, and 539 bp of POMC) from 113 individuals representing 20 species. The models with the best fit and the estimated parameters for each partition for the Bayesian analyses are shown in Table 2. Comparisons of partition strategies based on Bayes factors favored the 12-partition analysis (factors values > 200).

**Figure 1.**
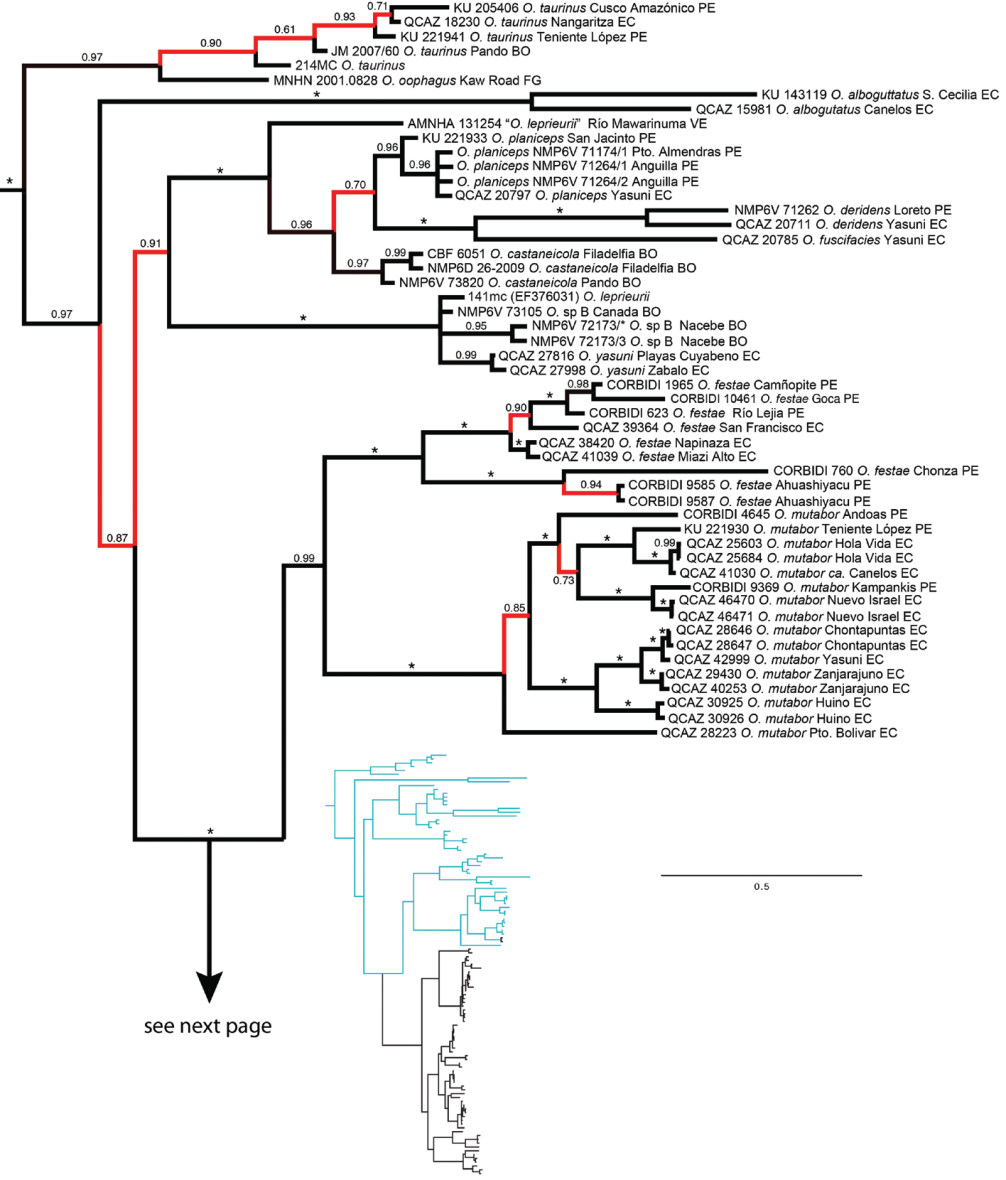
Bayesian consensus phylogram depicting relationships within *Osteocephalus*. Phylogram derived from analysis of 4170 bp of mitochondrial (gene fragments *12S*, *16S*, *ND1*, *CO1*, control region) and nuclear DNA (POM-C). Museum catalog no. (or, if unavailable, GenBank accession no.) and locality are shown for each sample. Posterior probabilities resulting from Bayesian Markov chain Monte Carlo searches appear above branches. An asterisk represents a value of 1 and red branches represent values < 0.95. Outgroup species (*Trachycephalus jordani* and T. *typhonius*)are not shown. Abbreviations are: **BO** Bolivia, **BR** Brazil, **CO** Colombia, **EC** Ecuador, **FG** French Guiana, **PE** Peru, **VE** Venezuela.

The topology (Fig. 1) is generally well supported and agrees with Salerno et al. (2012) phylogeny in showing a basal divergence between (*Osteocephalus taurinus* + *Osteocephalus oophagus*) and the other *Osteocephalus* species.Within the later, *Osteocephalus alboguttatus* diverges basally while the remaining species are divided in two clades. One clade (posterior probability, PP, = 1.0) corresponds to the *Osteocephalus buckleyi* species group. The other clade has weaker support (PP = 0.91) and consists of the phytotelmata breeding species (*Osteocephalus planiceps*, *Osteocephalus fuscifacies*, *Osteocephalus deridens*, *Osteocephalus castaneicola*; PP = 1.0) and *Osteocephalus leprieurii*, *Osteocephalus yasuni* and *Osteocephalus* sp. B (*sensu* Moravec et al. 2009; PP = 1.0). The “*Osteocephalus leprieurii*” sample in the clade with phytotelmata breeding is likely misidentified as suggested by Moravec et al. (2009).

All species within the *Osteocephalus buckleyi* species complex, except *Osteocephalus buckleyi*, are monophyletic. Individuals assignable to *Osteocephalus buckleyi* are paraphyletic relative to *Osteocephalus verruciger* and *O. cabrerai* and are separated in four clades (named A–D in Fig. 1).

Populations of *Osteocephalus mutabor* segregate latitudinally: the most divergent population (Puerto Bolívar) is the only north of the Napo and Aguarico rivers; the remaining populations are separated in one central and one southern clade, both with strong support. Pairwise genetic distances between populations are below 2% in all comparisons.

**Figure 2.**
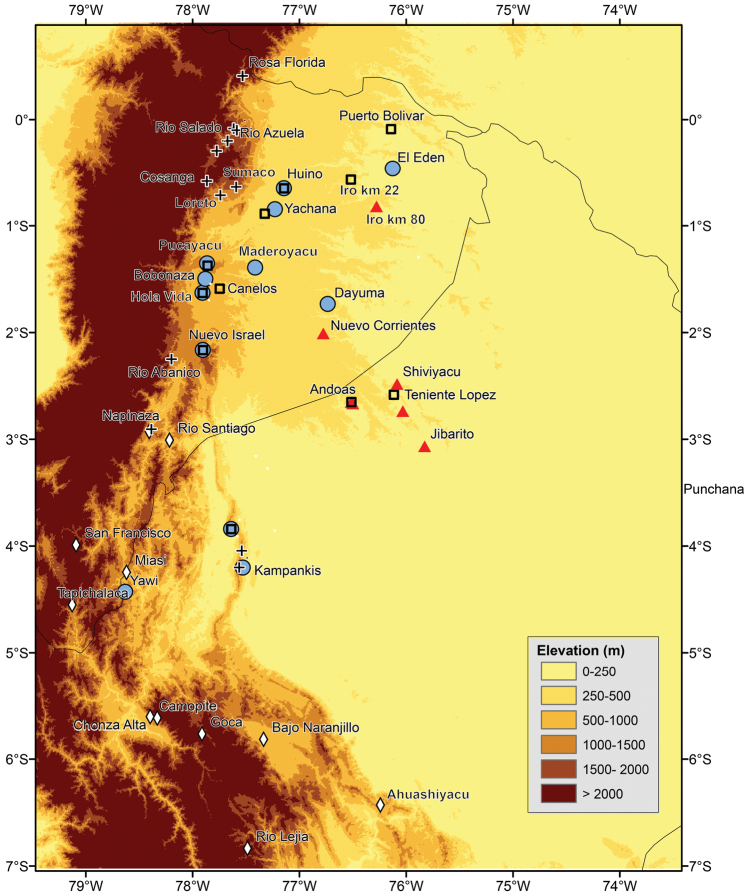
Records of *Osteocephalus cannatellai*, *Osteocephalus festae*, *Osteocephalus mutabor*, *Osteocephalus verruciger*, and *Osteocephalus vilmae*. *Osteocephalus cannatellai*, circles; *Osteocephalus festae*, diamonds; *Osteocephalus mutabor*, squares; *Osteocephalus verruciger*, crosses; and *Osteocephalus vilmae*, triangles. Locality data from the literature (Duellman and Mendelson 1995; Jungfer 2010; Peracca 1904; Ron et al. 2010) and specimens deposited at Museo de Zoología of Pontificia Universidad Católica del Ecuador, the Herpetology Collection, Escuela Politécnica Nacional, and Centro de Ornitología y Biodiversidad CORBIDI.

The phylogeny recovers a monophyletic *Osteocephalus verruciger* (in contrast to Ron et al. 2010) divided in two clades with an unexpected geographic pattern. Loreto and Pacto Sumaco are at a distance of 20–50 km from Cosanga, Río Salado and other nearby localities in central Ecuador (Fig. 2). Yet, in they phylogeny the two samples are sister to samples from Cordillera Kampankis in Peru, at a distance of 370 km. Cordillera Kampankis is an isolated mountain range separated from the rest of the Andes by areas below 500 m above sea level. The records from Cordillera Kampankis are the first confirmed occurrences of *Osteocephalus verruciger* in Peru. Genetic distances among *Osteocephalus verruciger* samples range between 0 and 1.5%.

*Osteocephalus festae* samples were collected on both sides of the dry valley of the Marañón River. This valley, with elevations as low as 600 m, is part of the Huancabamba depression, a well-known biogeographic barrier in the Andes. Nevertheless, populations on both sides do not form reciprocally monophyletic groups. In some cases, low genetic distances (e.g., San Francisco-Camñopite *p*-distance 0.3%) separate populations across the valley indicating recent gene flow. Relatively high genetic distances separate populations south of the Marañón (up to 2.8% between Catarata Ahuashiyacu and Camñopite).

**Figure 3.**
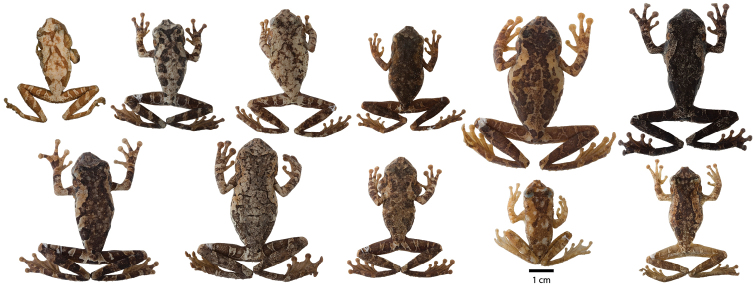
Variation in dorsal coloration of preserved specimens of adult *Osteocephalus buckleyi*. Left to right, upper row: BMNH 1947.2.13.44 (Lectotype), QCAZ 38704, EPN-H 6374, 11718 (males), QCAZ 2876, 14948 (females); lower row: QCAZ 39799, 26552 (females), 26488, 26561, 39364 (males). Provincia Napo, Orellana, Pastaza and Sucumbíos, Ecuador (See Appendix I for locality data). All specimens are shown at the same scale.

**Figure 4.**
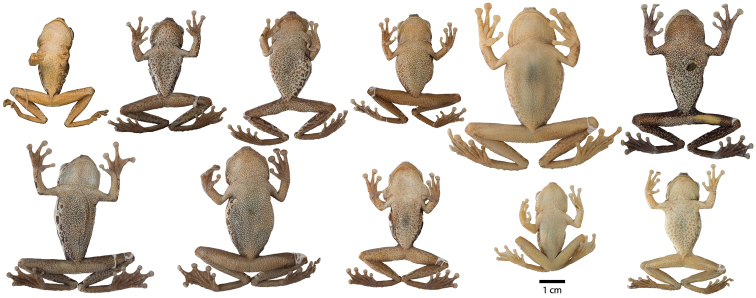
Variation in ventral coloration of preserved specimens of adult *Osteocephalus buckleyi*. Specimen identity and arrangement is the same as in Figure 3. All specimens are shown at the same scale.

**Figure 5.**
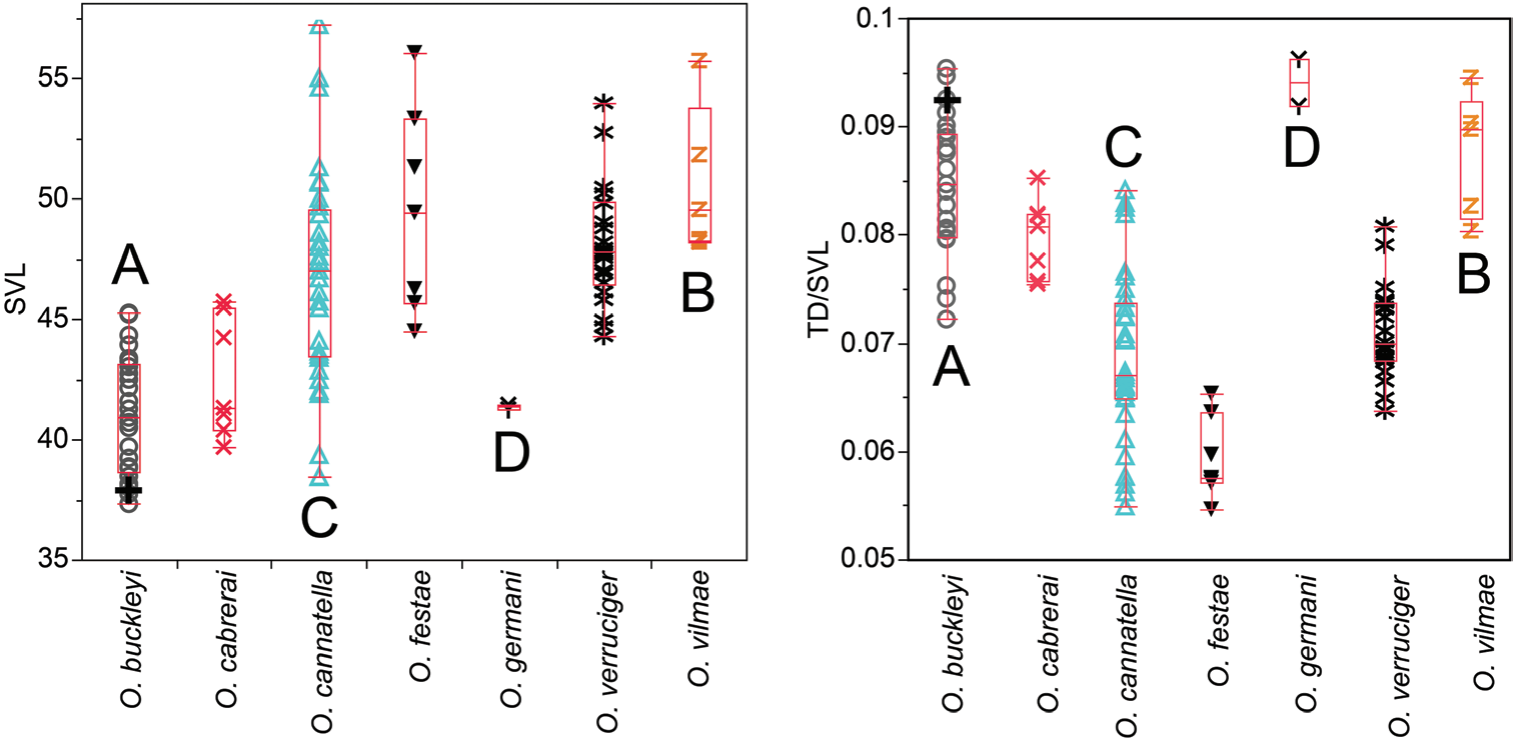
Boxplots for snout-vent length (SVL; left) and the ratio tympanum diameter/snout-vent length (TD/SVL; right). The line in the middle of the box represents the median, and the lower and upper ends of the box are the 25% and 75% quartiles respectively. Each individual is shown with a symbol; the cross in *Osteocephalus buckleyi* represents the lectotype. Letters correspond to those of clades on Figure 1.

**Figure 6.**
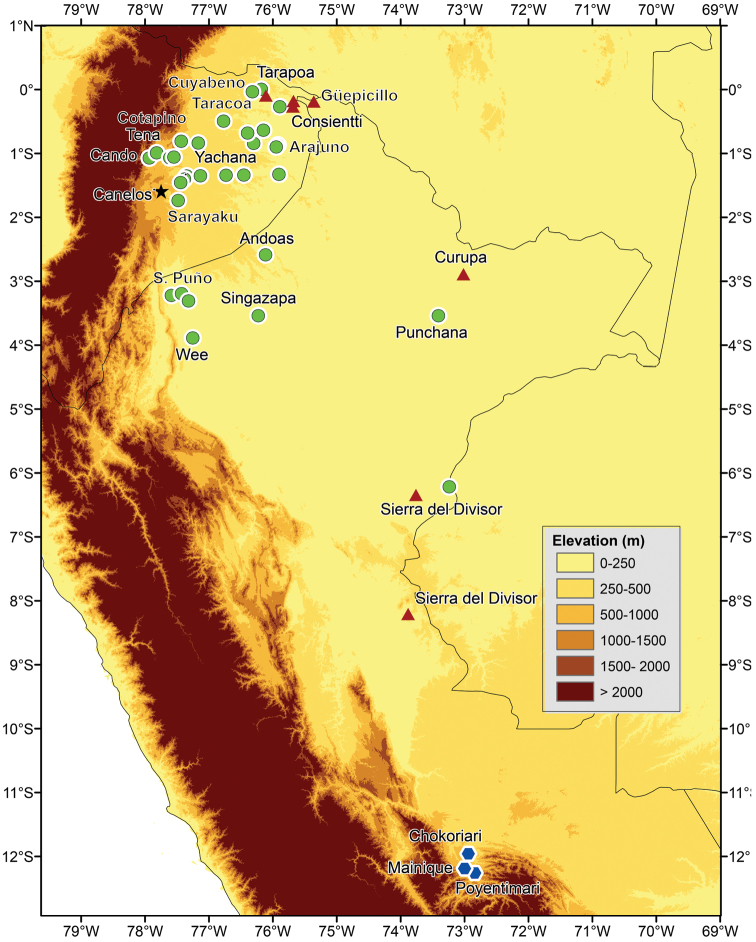
Records of *Osteocephalus buckleyi*, *Osteocephalus cabrerai*,and *Osteocephalus germani*. *Osteocephalus buckleyi*, circles; *Osteocephalus cabrerai*, triangles, and *Osteocephalus germani*, hexagons. The type locality of *Osteocephalus buckleyi* is shown with a star. Locality data from the literature (Duellman and Mendelson 1995; Jungfer 2010; Peracca 1904; Ron et al. 2010) and specimens deposited at Museo de Zoología of Pontificia Universidad Católica del Ecuador, the Herpetology Collection, Escuela Politécnica Nacional, and Centro de Ornitología y Biodiversidad CORBIDI.

*Osteocephalus buckleyi*-like individuals are grouped in four clades (A, B, C, and D in Fig. 1). Each clade has unique morphological features (see species descriptions) indicating that each represents a species. The external morphology of the lectotype of *Osteocephalus buckleyi* (BMNH 1947.2.13.44, an adult male with nuptial excrescences, Figs 3–4) shows that it belongs to clade A because: (1) its body size (37.90 mm; Fig. 5) is within the range for adult males of Clade A (37.32–45.25 mm, *n =* 24) but below de range of clades B (48.23–51.85, *n =* 4) and C (38.47–57.21 mm, *n =* 24), (2) its relative tympanum size (tympanum diameter/SVL = 0.093; Fig. 5) falls outside the range of Clade C (0.056–0.084, *n =* 24 males) but within the range of clade A (0.072–0.095, *n =* 24 males), (3) it has conspicuous tarsal tubercles (absent in clade D), and (4) clade D have a geographic range that, according to the available specimens, does not overlap with the type locality (Canelos, Provincia de Pastaza, Ecuador, 650 m; Figs 2 and 6). Thus, we attach the binomial *Osteocephalus buckleyi* to clade A. Clades B, C, and D cannot be assigned to any described species of *Osteocephalus* and thus represent new species that we describe herein as *Osteocephalus cannatellai* sp. n. (Clade C), *Osteocephalus germani* sp. n. (part of Clade D), and *Osteocephalus vilmae* sp. n. (Clade B).

Samples of *Osteocephalus buckleyi* *sensu stricto* (clade A) have low genetic differentiation (uncorrected *p* from 0 to 0.7%) despite including localities separated by up to 450 km. As in *Osteocephalus mutabor*, the most divergent populations in the phylogeny were those north of the Napo and Aguarico rivers (Cuyabeno and Tarapoa).

*Osteocephalus cannatellai* sp. nov comprises eight populations with genetic distances ranging from 0 to 1.7%. Populations group latitudinally forming a central and a southern clade. However, one of three samples (CORBIDI 9394) from the southern locality Pongo de Chinim (Kampankis) groups with the central localities.

Clade D comprises five samples from four populations. For two individuals (from Brazil and French Guyana) only GenBank sequences were available and thus we cannot determine if they belong to *Osteocephalus germani* sp. n. The three remaining samples (from Peru) are assigned to *Osteocephalus germani*.

**Table 2.** Post burn-in averages for parameters of Bayesian analyses. Abbreviations are: I = proportion of invariant sites, G = shape parameter of the gamma distribution of rate variation.

**Partition**	**Best-fit model**	**I**	**G**	**Rate Matrix**						**Base Frequency**			
				**AC**	**AG**	**AT**	**CG**	**CT**	**GT**	**A**	**C**	**G**	**T**
*12S*	GTR+G	–	0.201	0.049	0.347	0.071	0.028	0.494	0.009	0.330	0.249	0.182	0.238
*16S*	SYM+I+G	0.592	1.229	0.079	0.209	0.123	0.022	0.547	0.019	–	–	–	–
*CO1*, 1^st^ position	K80+I		–	–	–	–	–	–	–	–	–	–	–
*CO1*, 2^nd^ position	F81	–	–	–	–	–	–	–	–	0.168	0.269	0.155	0.408
*CO1*, 3^rd^ position	GTR+G	–	2.972	0.024	0.651	0.027	0.027	0.236	0.035	0.272	0.324	0.093	0.310
Control region	HKY+G	–	0.367	–	–	–	–	–	–	0.399	0.183	0.085	0.333
*ND1*, 1^st^ position	HKY+G	–	0.198	–	–	–	–	–	–	0.319	0.254	0.180	0.247
*ND1*, 2^nd^ position	HKY+I+G	0.716	0.043	–	–	–	–	–	–	0.177	0.290	0.126	0.407
*ND1*, 3rd position	GTR+G	–	1.780	0.032	0.601	0.036	0.018	0.295	0.019	0.353	0.273	0.098	0.275
POMC, 1^st^ position	F81+G	–	0.128	–	–	–	–	–	–	0.412	0.169	0.259	0.160
POMC, 2^nd^ position	F81	–	–	–	–	–	–	–	–	0.419	0.183	0.199	0.199
POMC, 3^rd^ position	HKY+I+G	0.485	0.761	–	–	–	–	–	–	0.318	0.319	0.164	0.199

### Species accounts

#### Osteocephalus cannatellai sp. n.

urn:lsid:zoobank.org:act:EDEC6BF4-F11C-4812-A06E-065C9035999D

http://species-id.net/wiki/Osteocephalus_cannatellai

##### Holotype.

**Figure 7.**
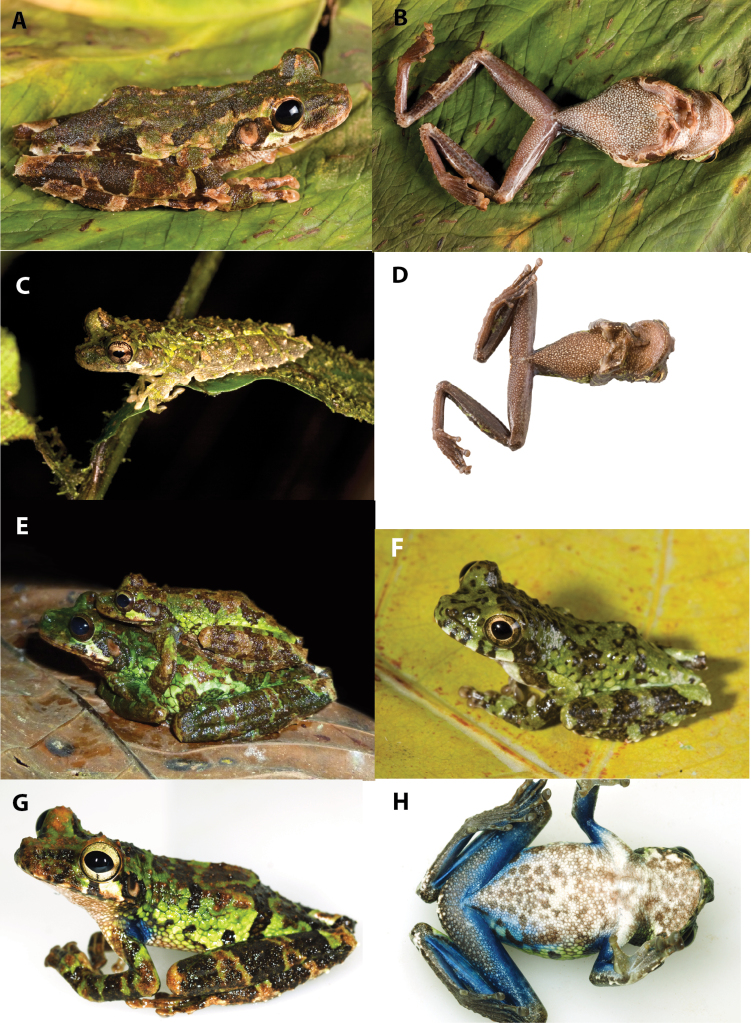
Dorsolateral and ventral views of *Osteocephalus*. **A–B** *Osteocephalus buckleyi*, QCAZ 43071, adult female, SVL = 50.95 mm, Jatun Sacha, Ecuador **C–D** *Osteocephalus cannatellai* QCAZ 48744, adult male, SVL = 51.96 mm, Reserva Zanjarajuno, Río Pucayacu, Ecuador **E** *Osteocephalus cannatellai*, amplectant pair QCAZ 49572 (holotype) adult male, SVL = 52.85 mm, from Río Pucayacu, Provincia Pastaza, Ecuador **F** *Osteocephalus cannatellai* QCAZ 40859, juvenile, SVL = 26.7 mm (type locality) **G–H** *Osteocephalus cannatellai* (CORBIDI 10534) from Cordillera Kampankis, Peru. Photographs **E** by R. Tarvin and **G–H** by A. Catenazzi.

**Figure 8.**
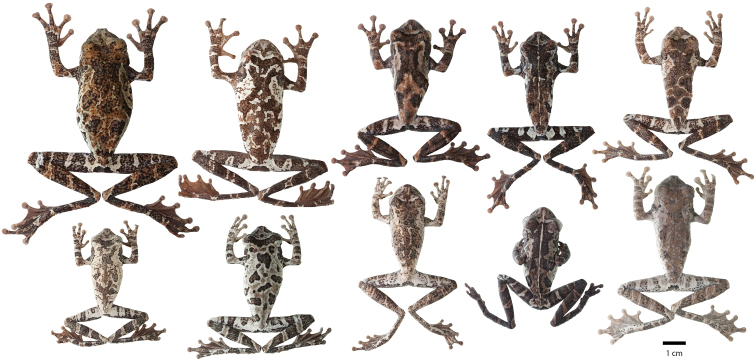
Adult *Osteocephalus cannatellai* showing variation in dorsal coloration of preserved specimens. Left to right, upper row (dark morphs): QCAZ 49439, 31051 (females), 40258, 49022, 45271 (males); lower row (light morphs): QCAZ 37175, 48744, 48797, 39633, 46472 (males). Ecuador, Provincia Morona Santiago, Napo, Orellana, Pastaza and Zamora Chinchipe. All specimens are shown at the same scale.

**Figure 9.**
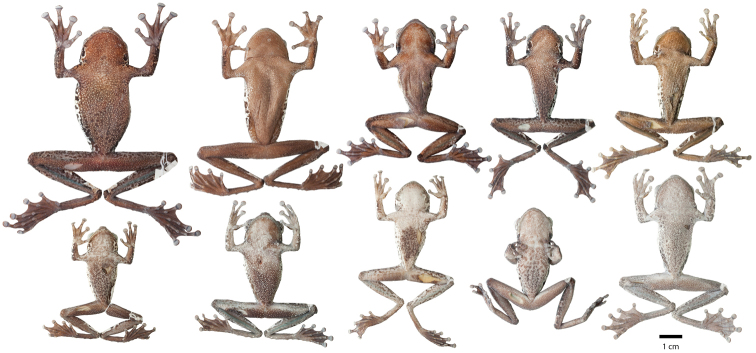
Adult *Osteocephalus cannatellai* showing variation in ventral coloration of preserved specimens. Specimen identity and arrangement is the same as in Figure 8. All specimens are shown at the same scale.

**Figure 10.**
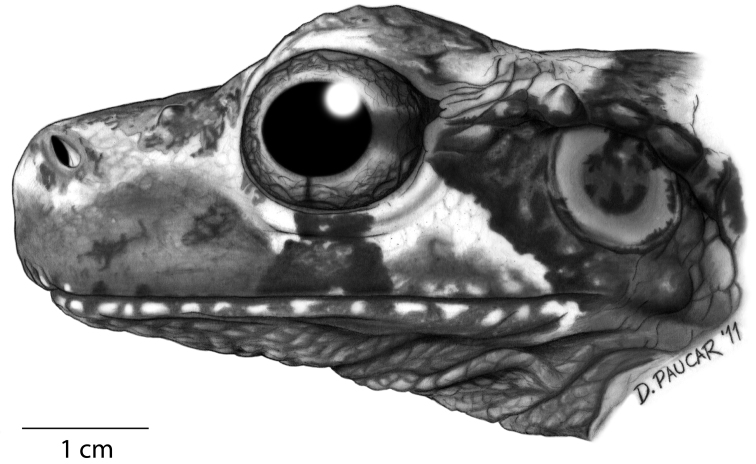
Lateral view of the head of the holotype of *Osteocephalus cannatellai* (QCAZ 49572).

(Figs 7–10) QCAZ 49572 (field no. PUCE 18835), adult male from Ecuador, Provincia Pastaza, Cantón Santa Clara, Río Pucayacu, in the vicinities of the Zanjarajuno Reserve (1.3578°S, 77.8477°W), 940 m above sea level, collected by P. Peña-Loyola, N. Peñafiel, and R. Tarvin on 3 July 2010.

##### Paratopotypes.

20 adult males, 1 adult female. QCAZ 33256, adult male, collected by I. G. Tapia, D. Almeida-Reinoso and M. Páez on 30 March 2007; QCAZ 39579, 39586–87, adult males, collected by D. Salazar-Valenzuela and G. Diaz between 12 and 14 December 2008; QCAZ 40909–10, adult males, collected by I. G. Tapia, L. A. Coloma, and S. R. Ron on 31 March 2008; QCAZ 40252, 40258, adult males, collected by D. Salazar-Valenzuela, D. Acosta-López and C. Korfel between 23 February and 1 March 2009; QCAZ 45271–72, 45277, 45281, adult males, collected by D. Acosta-López between 30 July and 2 August 2009; QCAZ 45907, 45909, adult males, collected by P. Peña-Loyola on 16 October 2009; QCAZ 49569–71, adult males, collected by N. Peñafiel between 26 June and 3 July 2010; QCAZ 49021–22, adult males, collected by R. Tarvin, and L. Bustamante on 3 August 2010; QCAZ 49439, adult female, collected by R. Tarvin and P. Aguilar on September 2010; QCAZ 48744 adult male, collected by S. R. Ron, L. Bustamante, I. G. Tapia , P. Peña-Loyola and R. Tarvin on 3 July 2010.

##### Paratypes.

42 adult males, 2 adult females. Ecuador: Provincia Morona Santiago: Bobonaza (1.4980°S, 77.8793°W), 660 m above sea level, QCAZ 32506, 32508, 32512, adult males, collected by L. A. Coloma and I. G. Tapia on 18 August 2008; Nuevo Israel (2.165°S, 77.902919°W), 1289 m above sea level, QCAZ 46472, adult male, collected by J. Brito-Molina on 2 January 2010; Provincia Napo: Reserva Yachana (0.8458°S, 77.2287°W), 300–350 m above sea level, QCAZ 48790, 48797, 48803–04, 48811, 48814, adult males, collected by S. North, S. Topp and G. Estevez between 4 June and 18 August 2008; Huino, QCAZ 50198, adult female, collected by W. C. Funk on February 2003; Provincia Orellana: El Edén (0.46147°S, 76.1252°W), 228 m above sea level, QCAZ 39633, adult male, collected by S. Aldás-Alarcón, Dayuma, Pozo Sunka (1.7333°S, 76.7333°W), 279 m above sea level, EPN-H 2752, 2755–56, 6372; Provincia Pastaza: Fundación Hola Vida (1.6285°S, 77.9072°W), 845 m above sea level, QCAZ 25607, 25469, adult males, collected by K. Elmer and I. G. Tapia on 27 June 2003, QCAZ 37175, adult male collected by I. G. Tapia, L. A. Coloma, P. Peña-Loyola and M. Páez on July 2007; Río Maderoyacu (1.3917°S, 77.4139°W), 500–600 m above sea level, EPN-H 6373, 6385, adult males, collected by A. Almendáriz; Provincia Zamora Chinchipe: Centro Shuar Yawi (4.4300°S, 78.6316°W), 945 m above sea level, QCAZ 31016, 31032–33, 31047, 31053, adult males, QCAZ 31051, adult female, collected by D. Almeida-Reinoso and A. Armijos between 13 and 19 September 2003. Peru: Región Loreto: Provincia Datem del Marañón: Cordillera de Kampankis: Pongo de Chinim (3.1130°S, 77.7762°W), 365 m above sea level, CORBIDI 09368, 09370, 09394, 9396, 10534, 10537, MUSM 28050, adult males collected by P. J. Venegas and A. Catenazzi on 3 August 2011; Quebrada Kampankis (4.0431°S, 77.5412°W), 325 m above sea level, CORBIDI 09507, 10535, adult males collected by P. J. Venegas and A. Catenazzi on 13 August 2011; Quebrada Wee (4.2041°S, 77.5298°W), 310 m above sea level, CORBIDI 09545–46, 09553, 09569, 10532–33, 10535–36, MUSM 28051, adult males, collected by P. J. Venegas and A. Catenazzi on 18 August 2011.

##### Diagnosis.

Throughout this section, coloration refers to preserved specimens unless otherwise noted. *Osteocephalus cannatellai* is a medium-sized species of *Osteocephalus* having the following combination of characters: (1) size sexually dimorphic; maximum SVL in males 57.21 mm (*n =* 33), in females 70.88 (*n =* 3); (2) skin on dorsum bearing scattered tubercles in males, smooth in females; (3) skin on flanks areolate; (4) hand webbing formula varying from I basal II1½ —2^1^/_3_III2^+^—2IV to I basal II2^–^—3^–^III2^2^/_3_—2½IV; foot webbing formula varying from I1—2½II1—2III1^+^—2IV2½—1^+^V to I0^+^—1^–^II0^+^—1^+^III0^+^—1½IV1^–^—0^+^V; (5) dorsum varying from dark brown with light gray marks to cream with brown marks; (6) venter varying from light gray to dark brown with lighter dots and/or dark brown blotches; (7) cream suborbital mark present, clear labial stripe absent; (8) flanks cream with darker reticulations anteriorly and dark marks; (9) dermal roofing bones of the skull weakly exostosed; (10) in life, bones green; (11) in life, iris bronze with irregular reticulations; (12) paired vocal sacs small, located laterally, behind jaw articulation, (13) in life, juveniles with bronze iris, without pale elbows, knees, and heels; (14) larvae unknown.

**Figure 11.**
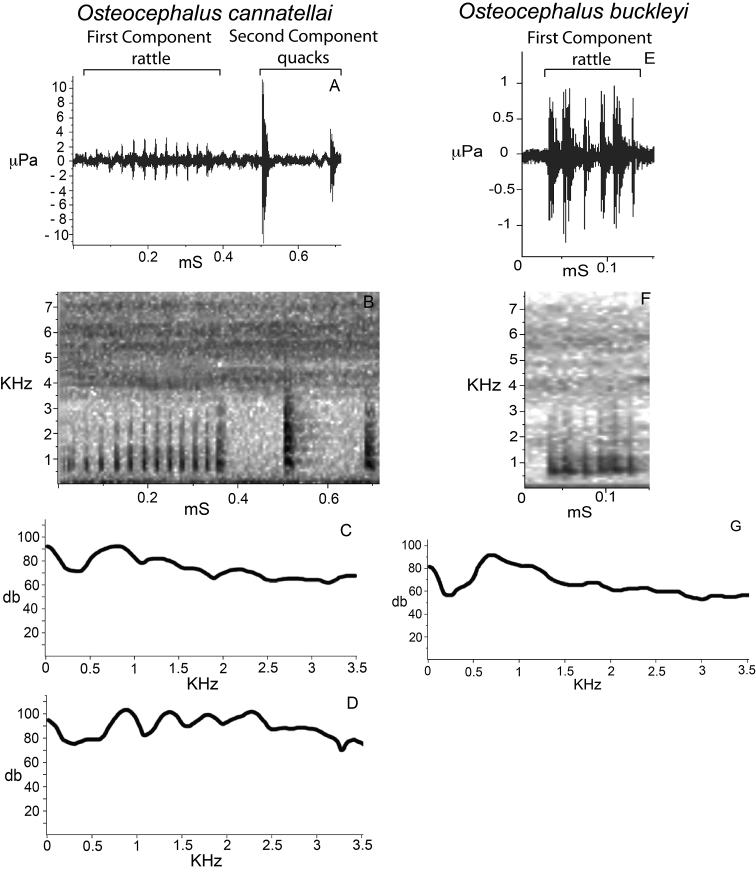
Advertisement calls of *Osteocephalus*. **A–D** *Osteocephalus cannatellai* (QCAZ8322) from Río Piraña, Provincia Orellana, Ecuador; **E–G** *Osteocephalus buckleyi*, from Jatun Sacha, Provincia Napo, Ecuador. **A** and **E** are oscillograms, **B** and **F** spectrograms, **C** and **G** power spectra of complete call, and **D** power spectra of quacks (second component).

*Osteocephalus cannatellai* is most similar to *Osteocephalus buckleyi* and *Osteocephalus vilmae* sp. n. The three species differ from other *Osteocephalus* by the combination of a bronze iris with irregular black reticulations (in life), areolate skin on the flanks, prominent tubercles in the tarsus and absence of a row of conspicuous tubercles in the lower jaw. *Osteocephalus cannatellai* differs from *Osteocephalus buckleyi* in having: (1) scattered and weakly keratinized dorsal tubercles (more abundant and keratinized in *Osteocephalus buckleyi*), (2) smaller tympanum (1/5 of head length in *Osteocephalus cannatellai* vs. 1/4 in *Osteocephalus buckleyi*; Fig. 5), (3) larger size (*Osteocephalus cannatellai* mean male SVL = 46.83, SD = 4.31, *n =* 33; *Osteocephalus buckleyi* mean male SVL = 41.12, SD = 2.45, *n =* 24; differences are significant: *t* = 5.82, *P* < 0.001; Fig. 5), (4) darker venter (cream with brown speckling in most *Osteocephalus buckleyi*; Figs 4 and 9.), (5) more extensive areolate area on flanks (from axillary region to groin in *Osteocephalus cannatellai*, restricted to anterior one third of flank in *Osteocephalus buckleyi*), (6) contrasting coloration between flanks and venter (change in coloration is gradual in *Osteocephalus buckleyi*), and (7) advertisement call (Fig. 11). Our phylogenetic analyses show that *Osteocephalus cannatellai* and *Osteocephalus buckleyi* are not sister species (Fig. 1).

**Figure 12.**
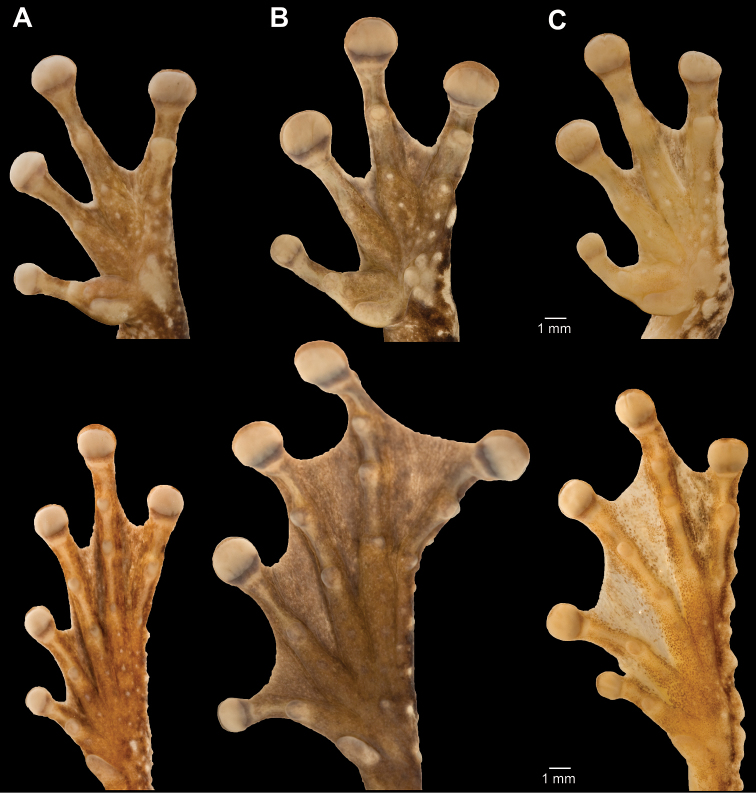
Ventral views of the left hand and foot of *Osteocephalus*. **A** *Osteocephalus buckleyi* (Tarangaro, Ecuador, SVL = 39.8 mm, QCAZ 39191) **B** *Osteocephalus cannatellai* (Zanjarajuno, Ecuador, SVL = 45.32 mm, QCAZ 45907)and **C** *Osteocephalus cabrerai* (Cuyabeno, Ecuador, SVL = 41.62 mm, EPN-H 7204). Hand and foot are shown at the same scale.

*Osteocephalus cannatellai* differs from *Osteocephalus vilmae* in having a narrower head (relative to SVL, mean male HW/SVL = 0.323, SD = 0.034, *n =* 33; *Osteocephalus vilmae* mean male HW/SVL = 0.355, SD = 0.012, *n =* 5; differences are significant: *t* = 2.06, *P* = 0.046) and a smaller tympanum (relative to SVL, mean male TD/SVL = 0.069, SD = 0.007, *n =* 33; *Osteocephalus vilmae* mean male TD/SVL = 0.087, SD = 0.006, *n =* 5; differences are significant: *t* = 5.17, *P* < 0.001). According to the phylogeny, *Osteocephalus cannatellai* and *Osteocephalus vilmae* are not sister species (Fig. 1). *Osteocephalus cannatellai* differs from *Osteocephalus cabrerai* in (1) lacking prominent tubercles on the lower jaw, (2) having smooth to tuberculate outer edge of Finger IV (outer edge with fringe in *Osteocephalus cabrerai*), and (3) having less webbing in the hands (in *Osteocephalus cannatellai* webbing reaches two thirds of the distance between the ultimate and penultimate tubercle of Finger IV, in *Osteocephalus cabrerai* it reaches the proximal border of the ultimate tubercle; Fig. 12). *Osteocephalus cannatellai* differs from other species of *Osteocephalus* (except *Osteocephalus buckleyi*, *Osteocephalus cabrerai*, and *Osteocephalus vilmae*)in having a combination of prominent tubercles in the tarsus and areolate skin in the flanks. A bronze iris with black reticulations further distinguishes *Osteocephalus cannatellai* from *Osteocephalus deridens*, *Osteocephalus oophagus*, *Osteocephalus planiceps*, and *Osteocephalus taurinus* which have black straight lines radiating from the pupil; iris coloration also differs in *Osteocephalus carri*, *Osteocephalus festae*, *Osteocephalus heyeri*, *Osteocephalus subtilis*, and *Osteocephalus verruciger* which have predominantly dark irises (Jungfer 2010; Jungfer and Lehr 2001; Lynch 2002). *Osteocephalus cannatellai* is larger than *Osteocephalus exophthalmus* (maximum male SVL in *Osteocephalus cannatellai* 57.21, *n =* 33; in *Osteocephalus exophthalmus* 32.7 mm, *n =* 3; Smith and Noonan 2001) and *Osteocephalus fuscifacies* (maximum SVL 44.17, *n =* 21). Skin texture in the flanks distinguishes *Osteocephalus cannatellai* (areolate) from *Osteocephalus mutabor* (smooth). *Osteocephalus inframaculatus* differs from *Osteocephalus cannatellai* in coloration of the ventral surfaces of hindlimbs (bold brown blotches in *Osteocephalus inframaculatus* are absent in *Osteocephalus cannatellai*; Jungfer 2010).

##### Description of holotype.

Adult male, 52.85 mm SVL, head length 18.61, head width 18.53, eye diameter 5.08, tympanum diameter 3.31, femur length 25.84, tibia length 30.05, foot length 22.73. Head narrower than body, slightly longer than wide; snout truncate in lateral and dorsal views; distance from nostril to eye longer than diameter of eye; canthus rostralis distinct and rounded; loreal region concave; internarial area depressed; nostrils moderately protuberant, directed laterally; interorbital area flat, lateral margins of the frontoparietals inconspicuous through skin; eye large, strongly protuberant; tympanic membrane clearly evident, large, slightly wider than high, about two thirds of eye diameter, separated from eye by ca. 85% of its diameter; tympanic annulus distinct except dorsally where it is covered by supratympanic fold; posterior end of supratympanic fold reaches arm insertion. Arm slender, axillary membrane present, reaching one third of arm length; four small low tubercles present along ventrolateral edge of forearm; relative length of fingers I<II<IV<III; fingers bearing large, oval discs, that of third finger about three fourths of tympanum diameter; subarticular tubercles prominent, round to ovoid, single; supernumerary tubercles present; palmar tubercle small, elongated; prepollical tubercle large, flat, elliptical; prepollex enlarged; large dark keratinous nuptial excrescences covering inner surface of prepollex up to half the distance between subarticular tubercle and proximal border of disk of thumb; webbing formula of fingers I basal II1^2^/_3_—2^2^/_3_III2½—2^+^IV. Medium sized to small tubercles on tibiotarsal articulation; scattered tubercles on tarsus, more abundant on outer edge; small tubercles scattered along ventrolateral edge of foot; toes bearing discs slightly wider than long, smaller than those of fingers; relative length of toes I<II<V<III<IV; outer metatarsal tubercle ill defined, small, round; inner metatarsal tubercle large, ovoid; subarticular tubercles single, round, protuberant; supernumerary tubercles restricted to the soles; webbing formula of toes I1—2II1—2^+^III1^+^—2^+^IV2^–^—1V. Skin on dorsum, head, and dorsal surfaces of limbs smooth, with scattered tubercles; skin on flanks areolate; skin on venter coarsely granular; skin on ventral surfaces of head and thighs granular, those of shanks smooth. Cloacal opening directed posteriorly at upper level of thighs; short simple cloacal sheath covering cloacal opening; round tubercles below vent; two conspicuous white tubercles ventrolateral to vent. Tongue cordiform, widely attached to floor of mouth; dentigerous processes of the vomer angular, adjacent medially, posteromedial to choanae, bearing 12 and 9 (left/right) vomerine teeth; choanae trapezoidal, oblique; vocal sac barely distinct above the arm and below the ear.

*Color of holotype in preservative*. Dorsum brown with light gray to cream peripheral marks; dark brown, ill defined, transversal bar between orbits (Fig. 8); cream middorsal line from tip of snout to end of sacrum; dorsal surfaces of forearms brown with light gray and dark gray marks, dorsal surfaces of thighs light gray with dark gray transversal bands, dorsal surfaces of shanks and feet brown with dark gray marks. Venter brown with light cream yellowish spots, more abundant on posterior half of the body (Fig. 9); ventral surfaces of hindlimbs and forelimbs brown with dark brown marks and conspicuous white tubercles on forearms; outer half of ventral surfaces of forearms dark brown; sides of head brown with oblique white bar from posteroventral border of orbit to border of jaw, below tympanum (Fig. 10); vertical dark brown bar below eye, anterior to white bar; area behind white bar and eye dark brown except for brown periphery of tympanum; iris light gray with black reticulations; flanks light gray anteriorly, cream posteriorly, areolate region with gray reticulation.

*Color of holotype in life*. Based on digital photographs. Dorsum brown with green peripheral marks; dark brown, ill defined, transversal bar between orbits (Fig. 7E); dorsal surfaces of forearms brown with green and dark brown marks, dorsal surfaces of thighs dark green with dark brown transversal bands, dorsal surfaces of shanks and feet brown with dark brown marks. Sides of head brown with oblique lime bar from posteroventral border of orbit to border of jaw, below tympanum; vertical dark brown bar below eye, anterior to lime bar; area posterior to lime bar and eye dark brown except for brown periphery of tympanum; flanks light green, areolate region with dark reticulation.

##### Etymology.

The specific name *cannatellai* is a noun in the genitive case and is a patronym for David C. Cannatella, who with his research has enriched the understanding of the evolution of Neotropical amphibians. He has also contributed to amphibian studies in Ecuador by providing funding and training to local scientists.

##### Variation.

Variation in dorsal and ventral coloration of preserved specimens is shown in Figures 8 and 9. Dorsal background coloration varies from cream to light gray or brown; irregular dark brown or dark gray marks are always present (Fig. 8). Some specimens have a cream middorsal line from the tip of the snout to the mid sacrum (QCAZ 49570) or the vent (QCAZ 39633). In females, the dorsum always lacks tubercles while in males it varies between lacking tubercles (QCAZ 32508) and having scant and ill-defined non-keratinized tubercles (e.g., QCAZ 48814 and 49569). The prominence of the tubercles seems to decrease in preserved specimens: when collected, QCAZ 48744 had large conspicuous dorsal tubercles (Fig. 7), in preservative tubercles are barely noticeable.

Ventral surfaces of preserved specimens (Fig. 9) vary from light gray (QCAZ 40909) to brown (QCAZ 31031). In most specimens, there are dark brown or dark gray spots, more abundant posteriorly (e.g., QCAZ 49439); QCAZ 39633 has brown blotches on the chest and venter; QCAZ 48804 has similar marks that also reach the gular region. In two Peruvian specimens ventral surfaces are light gray with few brown spots posteriorly (CORBIDI 09553) or with light brown spots, slightly visible, on gular region and belly (MUSM 28050). The gular regions in some Peruvian specimens are brown (e.g., CORBIDI 09507, 10532). Ventrally, limbs vary from light gray or light brown to dark brown; in QCAZ 33256 and 39587 black dots are present on limbs; scant cream tubercles can be present in the external edge of the forearm (e.g., QCAZ 32512). The skin of the anterior and posterior surfaces of thighs and the concealed surfaces of shanks are pale in the Peruvian specimens. The vent region is light gray to dark brown with dark brown dots. Flanks are cream to light gray, areolate in the anterior two-thirds and smooth posteriorly. In specimens from Peru the flanks are completely areolate. The areolate portion has a dark brown reticulation.

Head shape is truncate in dorsal and lateral view (e.g., QCAZ 39579). Lateral head coloration varies between dark brown (QCAZ 49569) to cream (QCAZ 32506). There is a cream subocular mark. The tympanic annulus is concealed dorsally and has lighter color than the background. The distal subarticular tubercle on Finger IV is single (e.g., QCAZ 40909) or bifid (e.g., QCAZ 45272).

Morphometric data pertain to adults and are summarized in Table 3.In the examined series, the largest male has a SVL of 57.21 mm and the largest female 72.77 mm; mean male SVL = 46.84 mm (*n =* 33, SD = 4.31), mean female SVL = 66.55 mm (*n =* 3, SD = 5.44). Females are significantly larger than males (*t* = 7.66, df = 33, *P* < 0.001). A MANOVA on the residuals of the regressions between SVL and the other measured variables indicates lack of significant differences between sexes in size-free morphometry (*F* = 0.239, df = 17, *P* = 0.060).

##### Color in life.

Based on digital photograph of adult male QCAZ 48744 (Fig. 7 C–D): dorsum green with irregular light and dark brown marks; canthal region green with cream subocular mark and olive green diffuse band along the posterior half of upper lip; tympanum light brown; flanks light green with dark brown reticulation anteriorly and irregular dark brown blotches posteriorly; dorsal surfaces of thighs, shanks and forelimbs green with transversal brown bands; venter brown with irregular dark brown and cream marks; iris bronze with diffuse brown mid-horizontal line and black reticulations.

Based on digital photograph of juvenile QCAZ 40859 (Fig. 7 F): dorsum green with dark brown marks; upper lip cream with transversal brown bars; flanks light green with brown marks; dorsal surfaces of arms, thighs and shanks green with brown transversal bars; external edge of tarsus with white tubercles; iris bronze with black reticulations and diffuse mid-horizontal dark band between the pupil and posterior border of iris.

In life the Peruvian specimens have extensive blue coloration in the groins, concealed surfaces of thighs and tibia, dorsal surfaces of tarsus, armpits and posterior surfaces of arms (e.g., CORBIDI 10534; Fig. 7 G–H). The iris is highly variable from light cream to brownish cream and dark brown (CORBIDI 09394); there are always black reticulations and a diffuse mid-horizontal dark band. Some individuals have a diffuse vertical dark band below the pupil.

Green coloration in life changes to cream in preserved specimens.

*Call*. Males call from vegetation next to rivers or streams. Acoustic parameters of the advertisement call of *Osteocephalus cannatellai* are shown in Table 4. The call consists of two components. The first is obligatory and consists of one to five rattle-like notes. The second component is facultative and consists of one to three quacks. The first component is pulsed and lacks harmonic structure; the second component has visible harmonics and reaches higher amplitude than the first component (Fig. 11).

The advertisement calls of *Osteocephalus cannatellai* differ markedly from those of *Osteocephalus buckleyi*. Calls of *Osteocephalus buckleyi* (Fig. 11) consist of a pulsed rattle–like note repeated at irregular intervals and without a second component. Those calls have a shorter duration, higher repetition rate, and fewer pulses than calls of *Osteocephalus cannatellai*.

##### Distribution and ecology.

*Osteocephalus cannatellai*has been recorded at twelve localities, all of them south of the Napo river, in the Ecuadorian and Peruvian Amazon basin (Provincias Morona Santiago, Napo, Orellana, Pastaza, Zamora-Chinchipe, and Datem del Marañón; Fig. 2). Localities with known elevation (El Edén, Huino, Yachana, Zanjarajuno, Río Maderoyacu, Hola Vida, Bobonaza, Nuevo Israel, Yawi, and Kampankis) have a range between 200 and 1290 m above sea level. Maximum airline distance between localities is 531 km. *Osteocephalus cannatellai* occurssympatrically with *Osteocephalus buckleyi* at Reserva Yachana and with *Osteocephalus fuscifacies* and *Osteocephalus mutabor* at Río Pucayacu, Nuevo Israel and Hola Vida.

Most specimens were collected at Río Pucayacu, a river surrounded by a mixture of primary and secondary forest. Frogs were found next to the river, perching over broad leaves or on tree branches 50 to 230 cm above the ground. At the collection site, the river has an average width of approximately 10 m, fast running water, and a rocky bottom. Males were calling next to the river between June 26 and July 3 2010. Several adults and a juvenile were found on a small stream, tributary of Río Rivadeneira, surrounded by secondary forest, near Río Pucayacu, in March 2008.

Vegetation types (according to the classification of Sierra et al. 1999 ) are: (1) Amazonian Mountain Range Evergreen Foothill Forest, characterized by a mixture of Amazonian and Andean vegetation with a canopy of 30 m (Río Pucayacu, Bobonaza, and Yawi), (2) Amazonian Lowland Evergreen Forests, characterized by high plant alpha-diversity and a canopy of 30 m with emergent trees that reach 40 m (Huino, Río Maderoyacu, Reserva Yachana), and (3) Amazonian Lower Montane Evergreen Forest, with an elevational range of 1300 to 2000 m above sea level, its canopy can reach 25 to 30 m (Nuevo Israel; Sierra et al. 1999, Cerón et al. 1999).

Specimens from Peru were collected in Cordillera de Kampankis within an elevational range of 300 to 365 m above sea level in tall, closed-canopy forest on low hills with well-drained soils at the base of the mountains. The soils have variable proportions of silt, clay and sand, but there are some small patches of sandy soil and limestone outcrops. The forest canopy is about 30 m tall, with emergent trees reaching 45 m. All individuals were collected in riparian vegetation of low-velocity and low-volume streams with rounded slate rocks lining the stream bed. Some individual were found on leafs of dense populations of rheophytic plants or shrubby *Pitcairnia aphelandriflora* (Bromeliaceae). Other individuals were found on branches of bushes between 50 and 200 cm above the ground. Other arboreal frogs at the site were *Osteocephalus mutabor*, *Hypsiboas cinerascens*, and *Gastrotheca longipes*.

#### Osteocephalus germani sp. n.

urn:lsid:zoobank.org:act:556B14DE-AA7D-4112-9C1B-D2814A7D6351

http://species-id.net/wiki/Osteocephalus_germani

##### Holotype.

**Figure 13.**
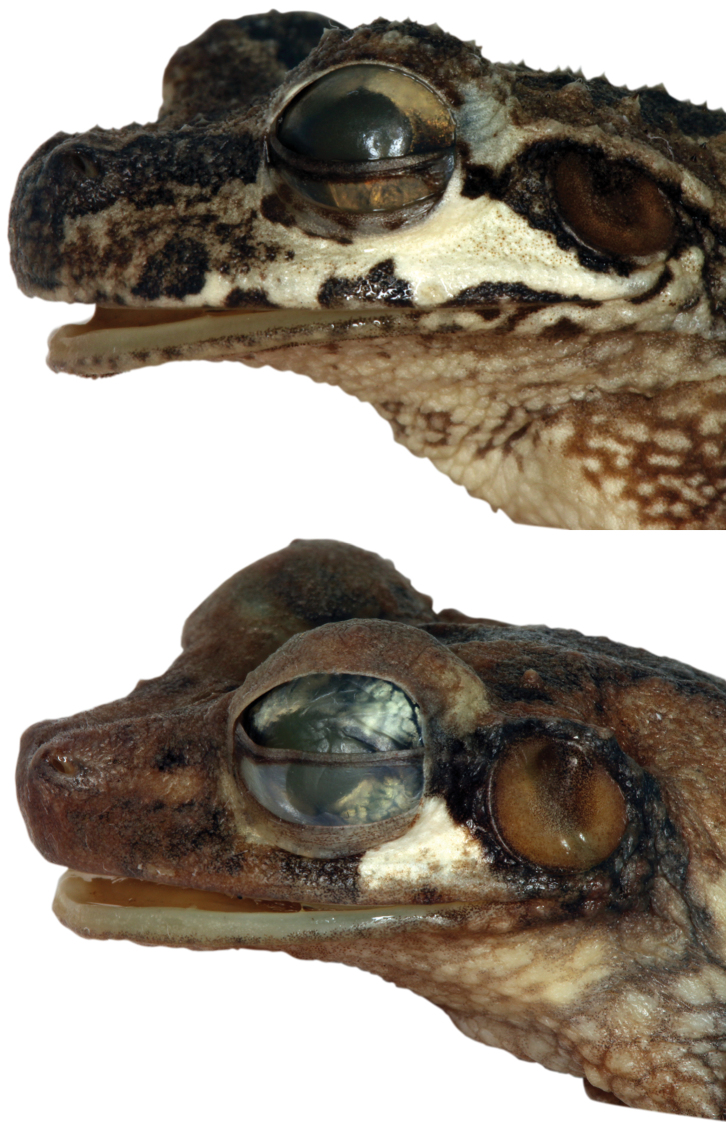
Lateral view of the head of the holotypes of *Osteocephalus germani* (above CORBIDI 05462) and *Osteocephalus vilmae* (below CORBIDI 04773).

**Figure 14.**
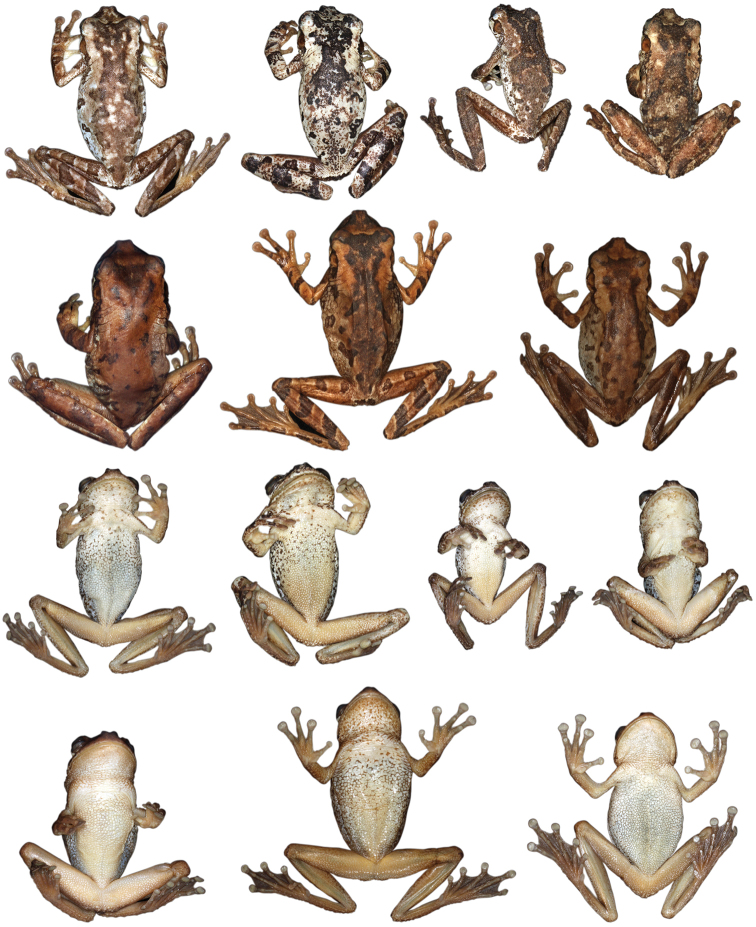
Adult *Osteocephalus germani* showing variation in dorsal and ventral coloration of preserved specimens. Left to right, upper row: CORBIDI 08267 (female), 05505 (female), 05462 (male, holotype), 06660 (male), CORBIDI 06663 (female), 08284 (female), 08059 (female); third and fourth rows show ventral views of the same specimens, in the same order as in the first two rows. Peru, Region Cusco.

(Fig. 13, 14) CORBIDI 05462, adult male from Peru, Región Cusco, Provincia La Convención, near Pongo de Mainique in the vicinity of Santuario Natural Megantoni (12.2581°S, 72.8425°W), 670 m above sea level, collected by G. Chavez on 23 April 2010.

##### Paratopotypes.

**Figure 15.**
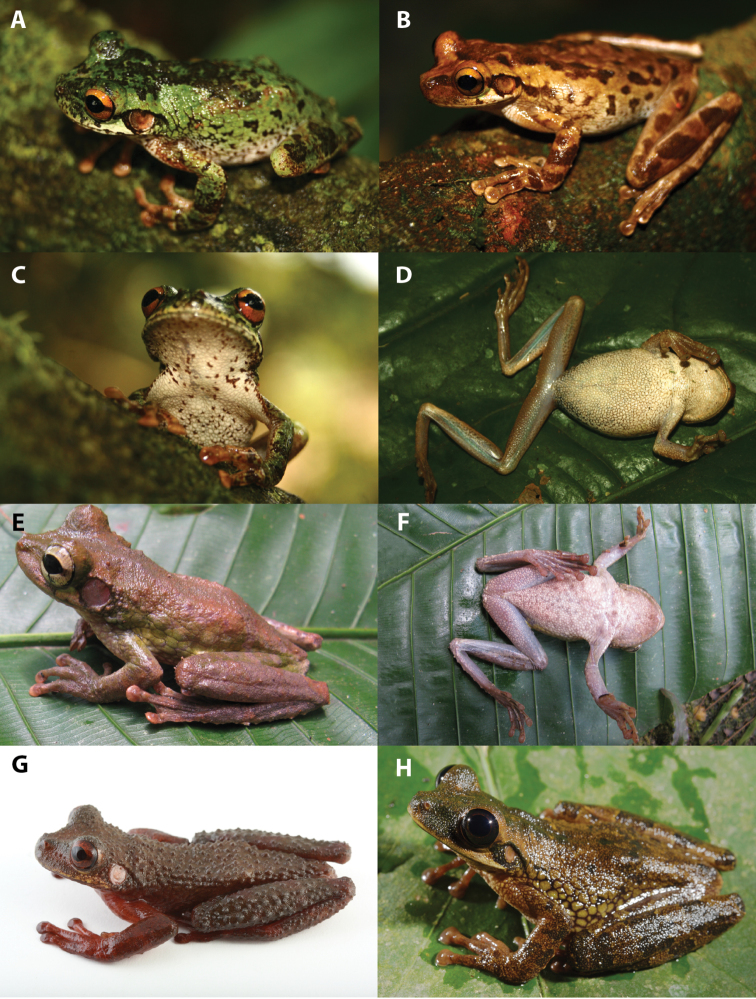
Dorsolateral, frontal, and ventral views of *Osteocephalus*. **A, C** *Osteocephalus germani*, CORBIDI 05505, adult female, SVL = 49.16 mm, Pongo de Mainique, Peru **B, D** *Osteocephalus germani*, CORBIDI 08284, adult female, SVL = 49.00 mm, Comunidad Nativa Poyentimari, Peru **E–F** *Osteocephalus vilmae* CORBIDI 04773 (holotype), adult male, SVL = 51.85 mm, Pampa Hermosa, Peru **G** *Osteocephalus verruciger*, QCAZ 41115, adult male, 52.37 mm, Pacto Sumaco, Ecuador **H** *Osteocephalus festae*, QCAZ 39801, adult female, SVL = 51.54 mm, Río Napinaza, Ecuador. Photographs **A–D** by G. Chavez, and **E–F** by V. Durán.

(Fig. 15 A, C) CORBIDI 06633, adult female, and CORBIDI 06660, adult male, collected with the holotype; CORBIDI 05505, adult female, collected by G. Chavez on 8 November 2009.

##### Paratypes.

(Fig. 15 B, D) Peru: Provincia La Convención, Comunidad Nativa Poyentimari (12.18853°S, 73.00092°W), 725 m above sea level, CORBIDI 08267, 08284, adult females, collected by G. Chavez and D. Vasquez on 28 November 2010; Comunidad Nativa Chokoriari (11.9569°S, 72.9410°W), 434 m above sea level, CORBIDI 08059, adult female, collected by D. Vasquez on 8 December 2010.

##### Diagnosis.

Throughout this section, coloration refers to preserved specimens unless otherwise noted. *Osteocephalus germani* is a medium-sized species of *Osteocephalus* having the following combination of characters: (1) size sexually dimorphic; maximum SVL in males 41.45 mm (*n =* 2), in females 50.76 (*n =* 2); (2) skin on dorsum bearing tubercles in males, smooth in females; (3) skin on flanks areolate; (4) hand webbing formula varying from I basal II2^–^—3^–^III2½—2IV to I basal II2—3III3^–^—3^–^IV; foot webbing formula varying from I1—1½II1^–^—2III1—2IV1½—1^–^V to I1^+^—2II1^+^—2III1^+^—2^+^IV2—1V; (5) dorsum varying from brown with dark brown marks to light gray with dark brown marks; (6) venter light cream with or without dark brown flecks; (7) cream suborbital mark present, clear labial stripe absent; (8) flanks cream to brownish cream with dark brown blotches and flecks; (9) dermal roofing bones of the skull weakly exostosed; (10) bones green in life; (11) in life, iris golden to reddish golden with fine dark reticulation; (12) paired vocal sacs, located laterally, behind jaw articulation, (13) juvenile coloration unknown; (14) larvae unknown.

**Figure 16.**
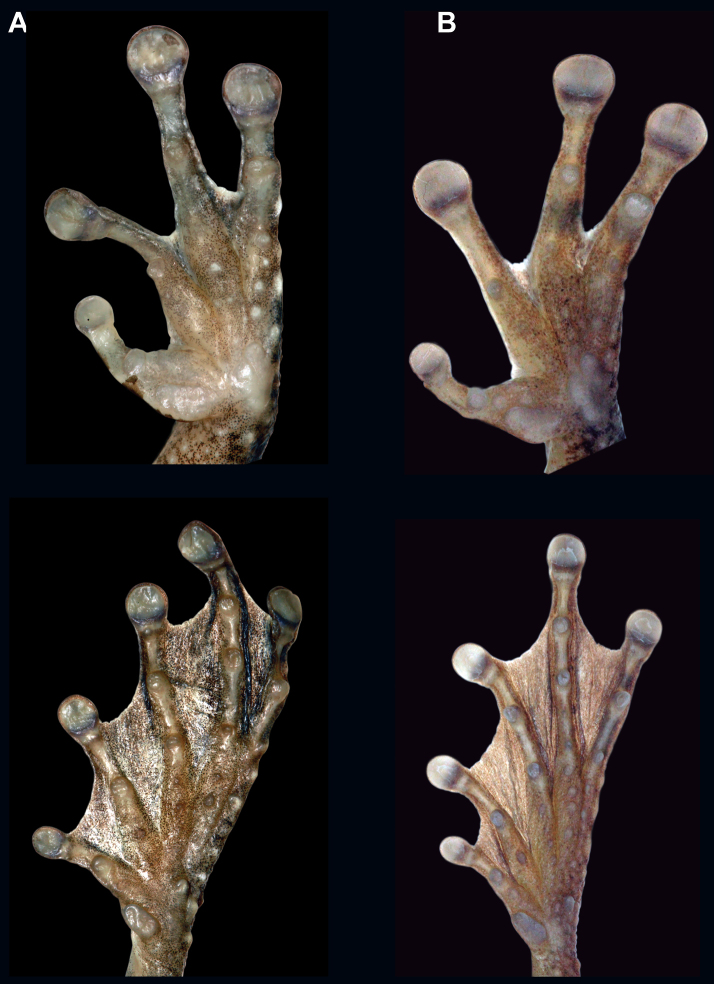
Ventral views of left hand and foot of *Osteocephalus vilmae* and *Osteocephalus germani*. **A** *Osteocephalus vilmae* (Jibarito, Peru, SVL = 48.31 mm, CORBIDI 06469), and **B** *Osteocephalus germani* (Comunidad Nativa Poyentimari, Peru, SVL = 49.00 mm, COBIDI 08284).

*Osteocephalus germani* is most similar to *Osteocephalus buckleyi*, *Osteocephalus cabrerai*, *Osteocephalus cannatellai* sp. n., and *Osteocephalus vilmae* sp. n. It differs from all of them in lacking prominent tarsal tubercles (tubercles are indistinct in *Osteocephalus germani*). It further differs from *Osteocephalus buckleyi*, *Osteocephalus cannatellai*, and *Osteocephalus vilmae* in having a white to light cream venter with or without dark brown flecks (cream with brown speckling in most *Osteocephalus buckleyi* and *Osteocephalus vilmae*,light gray to dark brown in *Osteocephalus cannatellai*). *Osteocephalus germani* also differs from *Osteocephalus cannatellai* and *Osteocephalus vilmae* in having more abundant and keratinized dorsal tubercles (dorsal tubercles are scattered and weakly keratinized in *Osteocephalus cannatellai* and *Osteocephalus vilmae*) and smaller size (*Osteocephalus germani* male SVL range = 41.26–41.45, *n =* 2; *Osteocephalus cannatellai* male SVL range = 38.46–57.21, *n =* 33; *Osteocephalus vilmae* male SVL range = 48.23–55.77, *n =* 5). Mitochondrial DNA sequences show that *Osteocephalus germani* is the sister species of *Osteocephalus cabrerai* (Fig. 1). *Osteocephalus germani* can be easily distinguished from *Osteocephalus cabrerai* by (*Osteocephalus cabrerai* in parenthesis): (1) absence of prominent tubercles on the lower jaw (present), (2) smooth outer edge of Finger IV (outer edge with fringe), (3) row of inconspicuous tubercles in the outer edge of tarsus (conspicuous), and (4) less webbing in the hands (in *Osteocephalus germani* web reaches the antepenultimate tubercle of Finger IV, in *Osteocephalus cabrerai* it reaches the proximal border of the ultimate tubercle; Figs 12 and 16). *Osteocephalus germani* differs from other species of *Osteocephalus* in having a combination of a dark golden to tan golden iris, a row of indistinct tubercles in the tarsus, and areolate skin in the flanks. A golden iris with black reticulations further distinguishes *Osteocephalus germani* from *Osteocephalus deridens*, *Osteocephalus oophagus*, *Osteocephalus planiceps*, and *Osteocephalus taurinus* which have bronze to golden irises with black lines radiating from the pupil; iris coloration also differs in *Osteocephalus carri*, *Osteocephalus festae*, *Osteocephalus heyeri*, *Osteocephalus subtilis*, and *Osteocephalus verruciger* which have predominantly dark irises, and in *Osteocephalus leoniae* which have a bicolor iris (Jungfer 2010; Jungfer and Lehr 2001; Lynch 2002). *Osteocephalus germani* differs from *Osteocephalus exophthalmus*, *Osteocephalus fuscifacies* and *Osteocephalus leoniae* in having abundant keratinized dorsal tubercles in males (tubercles are absent in the three last species). Skin texture in the flanks distinguishes *Osteocephalus germani* (areolate) from *Osteocephalus mutabor* (smooth). *Osteocephalus inframaculatus* differs from *Osteocephalus germani* in coloration of the ventral surfaces of hindlimbs (bold brown blotches in *Osteocephalus inframaculatus* are absent in *Osteocephalus germani*; Jungfer 2010).

##### Description of holotype.

Adult male, 41.26 mm SVL, head length 12.79, head width 14.23, eye diameter 5.23, tympanum diameter 3.79, femur length 22.3, tibia length 23.1, foot length 17.97. Head narrower than body, slightly wider than long; snout rounded in dorsal view and truncate in lateral view; distance from nostril to eye longer than diameter of eye; canthus rostralis distinct and straight; loreal region concave; internarial area depressed; nostrils moderately protuberant, directed laterally; interorbital area with tiny keratinized conical tubercles, lateral margins of frontoparietals inconspicuous through skin; eye large, strongly protuberant; tympanic membrane clearly evident, large, slightly wider than high, about two thirds of eye diameter, separated from eye by ca. 85% of its diameter; tympanic annulus distinct except dorsally where it is covered by supratympanic fold; posterior end of supratympanic fold reaches arm insertion. Arm slender, axillary membrane present, reaching less than one third of arm length; four small low tubercles present along ventrolateral edge of forearm; relative length of fingers I<II<IV<III; fingers bearing large, oval discs, that of third finger about three fourths of tympanum diameter; subarticular tubercles prominent, round to ovoid except for bifid distal subarticular tubercle of Finger IV; supernumerary tubercles present; palmar tubercle small, elongated; prepollical tubercle large, flat, elliptical; prepollex enlarged; large dark keratinous nuptial excrescences covering inner surface of prepollex up to two thirds the distance between subarticular tubercle and proximal border of disk of thumb; webbing absent between fingers I and II; webbing formula of fingers II2^–^—3III2½ —3^–^IV. Small tubercles on tibiotarsal articulation; dorsal surface of tarsus covered by tiny keratinized conical tubercles, more abundant on outer edge; minute tubercles scattered along ventrolateral edge of foot; toes bearing discs slightly wider than long, smaller than those of fingers; relative length of toes I<II<V<III<IV; outer metatarsal tubercle ill defined, small, round; inner metatarsal tubercle low, ovoid; subarticular tubercles single, round, protuberant; supernumerary tubercles restricted to the soles; webbing formula of toes I1—2II1—2III1^+^—2IV2^–^—1V. Skin on dorsum, head, and dorsal surfaces of limbs shagreen covered by conical tubercles with keratinized tips, tiny on head and limbs; skin on flanks areolate; skin on venter coarsely granular; skin on ventral surfaces of head and thighs granular, those of shanks smooth. Cloacal opening directed posteriorly at upper level of thighs; short simple cloacal sheath covering cloacal opening; round tubercles below vent; two conspicuous white tubercles ventrolateral to vent at lower level of thighs. Tongue cordiform, widely attached to floor of mouth; dentigerous processes of the vomers angular, adjacent medially, posteromedial to choanae, bearing 5 and 6 (left/right) vomerine teeth; choanae trapezoidal, oblique; vocal slits moderately long, extending diagonally from lateral end of tongue toward to the angle of snout; vocal sac indistinct above the arm and below the ear.

*Color of holotype in preservative*. Dorsum light brown with dark brown peripheral marks; dark brown transversal bar between orbits with fine pale borders; dorsal surfaces of forearms grayish brown with dark brown marks, dorsal surfaces of thighs, shanks, and feet grayish brown with diffuse brown transversal bars. Venter light cream with dark brown flecks on the throat and thoracic region and absent on posterior half of the body; ventral surfaces of hindlimbs and forelimbs dirty cream with dark brown flecks on the lateral borders of shanks; outer half of ventral surfaces of forearms dirty cream; sides of head brown with white subocular band extending, below tympanum, two little brown blotches below the eye; tympanic membrane dark brown and area in the periphery of tympanum light brown dorsally and grayish brown behind the tympanum; flanks grayish white, areolate region with dark brown reticulation and flecks. Iris silver with dark brown mid-horizontal line and thin black reticulations.

##### Color in life.

Dorsum brown with irregular dark brown marks; flanks brownish cream with dark brown spots and flecks; dorsal surfaces of thighs, shanks, and forelimbs brown with transversal dark brown bands. Venter whitish cream with brown flecks in throat; ventral surfaces of thighs tan. Iris bronze with thin black reticulations (G. Chávez field notes April 2010).

##### Etymology.

 The new species is dedicated to our colleague German Chávez (CORBIDI), one of the best friends of PJV, for his contributions to Peruvian herpetology and collecting the type series and tissues of this new species.

##### Variation.

Variation in dorsal and ventral coloration of preserved specimens is shown in Figure 14. Dorsal background coloration varies from light brown to light gray; irregular dark brown marks are always present. In females, the dorsum lacks tubercles while in males tubercles are present. The single male paratype (CORBIDI 06660) differs from the holotype in having non–keratinized tubercles.

Ventral surfaces of preserved specimens (Fig. 14) are whitish cream. All the specimens have scattered dark brown flecks on the anterior half of the venter. Ventrally, limbs vary from whitish cream to tan; scant white tubercles can be present in the external edge of the forearm of males (e.g., CORBIDI 06660). The vent region is light brown or dark. Flanks are whitish cream to light gray, areolate in the anterior half and nearly smooth posteriorly. The areolate portion is completely covered by dark brown reticulation and flecks.

Snout is truncate in lateral view except for a female with rounded snout (CORBIDI 06633). Lateral head coloration varies from dull brown (CORBIDI 06633) to cream with dark brown blotches (CORBIDI 05505). The tympanic annulus is concealed dorsally and has lighter color than the background. The distal subarticular tubercle on Finger IV is bifid in all the specimens.

Adult morphometric data are summarized in Table 3.In the examined series, the largest male has a SVL of 41.45 mm and the largest female 50.76 mm; mean male SVL = 41.35 mm (*n =* 2, SD = 0.13), mean female SVL = 49.96 mm (*n =* 2, SD = 1.13).

##### Color in life.

Based on digital photograph of adult male CORBIDI 06660: dorsum brown with irregular dark brown marks and some scattered light green blotches; canthal region greenish brown with greenish cream subocular mark and dark labial bars; tympanum light brown; flanks light green with dark brown reticulation and dark brown blotches posteriorly; dorsal surfaces of thighs, shanks, and forelimbs brown with transversal dark brown bands and scattered light green blotches. Iris bronze with brown horizontal midline and thin black reticulations.

Based on digital photograph of adult female CORBIDI 06633: dorsum brown with few scattered irregular dark brown marks; canthal region dark brown with greenish cream subocular mark speckled by three small dark brown blotches; tympanum light brown; flanks light brown with dark brown blotches; ventrolateral region cream with fine dark reticulation; dorsal surfaces of thighs, shanks, and forelimbs brown with transversal dark brown bands. Anterior half of venter whitish cream with fine brown reticulation in throat and chest; posterior half of venter and ventral surfaces of thighs tan; iris bronze with diffuse brown mid-horizontal line and thin black reticulations.

Based on digital photograph of adult female CORBIDI 05505 (Fig. 15): dorsum green with irregular dark brown marks; canthal region green with brown mottling and white subocular mark extending to the lips as a white labial stripe along posterior half of the jaw; tympanum light brown; flanks white with dark brown reticulation and small dark brown blotches posteriorly; dorsal surfaces of thighs, shanks, and forelimbs green with transversal dark brown bands and flecks. Venter white with scattered brown flecks on throat and chest. Iris reddish gold with diffuse brown mid-horizontal line and thin black reticulations. Based on digital photograph of adult female CORBIDI 08284 (Fig. 15): dorsum light brown with irregular dark brown marks; canthal region brown and greenish white subocular mark; tympanum light brown; flanks light brown with small dark brown blotches; dorsal surfaces of thighs, shanks, and forelimbs light brown with transversal dark brown bands. Venter dull cream. Iris bronze with diffuse brown mid-horizontal line and thin black reticulations.

##### Distribution and ecology.

*Osteocephalus germani* is known from three localities in southern Peru (Fig. 6). Pongo de Manique and Comunidad Nativa de Poyentimari are in premontane forest on the Upper Urubamba River basin (vegetation types according to ONERN 1976) in the Amazonian foothills of the southern Peruvian Andes, at elevations of 670–725 m; Comunidad Nativa de Chokoriari is *Terra Firme* Amazonian lowland forests on the lower Urubamba River basin in the southern Peruvian Amazon lowlands, at elevation of 434 m. In Pongo de Mainique the new species was found close to rocky streams in low-hill primary forest with arboreal ferns and abundant epiphytes. At this locality, *Osteocephalus germani* was sympatric with *Osteocephalus castaneicola* and *Osteocephalus mimeticus*. In Comunidad Nativa de Poyentimari, *Osteocephalus germani* was found close to rocky streams in a step area of very wet high-hill primary forest with abundant ferns (including arboreal), epiphytes, lichens and mosses. At this locality the new species was sympatric with *Osteocephalus mimeticus*. In Comunidad Nativa de Chokoriari, *Osteocephalus germani* was found close to a black-water slow-running creek in a patch of secondary forest, surrounded by pastures for cattle and plantations. The forest was dominated by bamboo and *Cecropia* spp. and the creek had sandy soils covered by leaf litter. No other species of *Osteocephalus* were found in this locality.

All specimens were collected next to temporary pools, perching over broad leaves or on tree branches 100 to 200 cm above the ground. Many streams surround the collection sites.

##### Remarks.

In the phylogeny (Fig. 1), two specimens from gen bank (EF376030 from French Guiana and AY843705 from Río Jurua, Brazil) are grouped with *Osteocephalus germani* in a strongly supported clade (PP = 0.96) and are likely conspecific or represent one or two closely related species. The specimen from French Guiana was reported as “*Osteocephalus oophagus*” by Salducci et al. (2002, 2005); the specimen from Brazil was reported as “*Osteocephalus cabrerai*” by Faivovich et al. (2005). Both individuals appear to be misidentified.

#### Osteocephalus vilmae sp. n.

urn:lsid:zoobank.org:act:681AAC6A-8710-4276-AA79-BD1F5C58C1DC

http://species-id.net/wiki/Osteocephalus_vilmae

##### Holotype.

(Figs 13 and 15) CORBIDI 04773, adult male from Peru, Region Loreto, Provincia Datem del Marañón, Pampa Hermosa (3.0650°S, 75.8264°W), 200 m above sea level, collected by V. Duran on 28 March 2008.

##### Paratypes.

Five adult males: Ecuador: Provincia de Orellana: Pompeya-Iro road, km 80, Río Beye, QCAZ 51205, collected by E. Toral, I. G. Tapia, T. Camacho, and S. R. Ron on 31 May 2011; Provincia Pastaza: Nuevo Corrientes, 250 m above sea level, QCAZ 14947, collected by F. Villamarín on August 2000. Peru: Provincia Datem del Marañón: Andoas (2.6516°S, 76.5137°W), 151 m above sea level, CORBIDI 01086, collected by A. Delgado on September 2008; Jibarito (2.7356°S, 76.0318°W), 197 m above sea level, CORBIDI 06469, collected by A. Delgado on 14 July; Capihuari Norte (2.6642°S, 76.5012°W), 270 m above sea level, CORBIDI 05031, collected by J. C. Chaparro on March 2008.

##### Diagnosis.

Throughout this section, coloration refers to preserved specimens unless otherwise noted. *Osteocephalus vilmae* is a medium-sized species of *Osteocephalus* having the following combination of characters in males (females are unknown): (1) maximum SVL in males 55.77 mm (*n =* 6); (2) skin on dorsum bearing few scattered to abundant tubercles; (3) skin on flanks areolate with big flattened warts; (4) hand webbing formula varying from I basal II basal III2^–^—2IV to I basal II1^2^/_3_—2^2^/_3_III2^2^/_3_—2½IV; foot webbing formula varying from I1—1½II1—2^–^III1^–^—2IV2^–^—1^–^V to I1^+^—2 II1—2III1^+^—2^+^IV2—1^+^V (Fig. 16); (5) dorsum varying from light brown with dark brown marks to light gray with dark brown marks; (6) venter varying from light gray to tan with lighter dots and/or dark brown blotches; (7) cream suborbital mark present, clear labial stripe absent; (8) flanks cream with darker reticulations and dark marks; (9) dermal roofing bones of the skull weakly exostosed; (10) in life, bones green; (11) in life, iris light cream to dirty cream with irregular reticulations; (12) paired vocal sacs small, located laterally, behind jaw articulation, (13) juveniles unknown; (14) larvae unknown.

*Osteocephalus vilmae* is most similar to *Osteocephalus buckleyi* and *Osteocephalus cannatellai*. Itdiffers from *Osteocephalus buckleyi* in having (1) scattered and weakly keratinized dorsal tubercles (abundant and keratinized in *Osteocephalus buckleyi*), (2) larger size (*Osteocephalus vilmae* mean male SVL = 55.77, SD = 3.17, *n =* 5; *Osteocephalus buckleyi* mean male SVL = 41.12, SD = 2.49, *n =* 24; differences are significant: *t* = 6.50, *P* < 0.001; Fig. 5), and (3) more extensive and conspicuous areolate area on flanks (from axillary region to groin, with big flattened warts, in *Osteocephalus vilmae*; restricted to anterior one half of flank in *Osteocephalus buckleyi*). The range of genetic distances (uncorrected *p* for gen 12S) between *Osteocephalus vilmae* and *Osteocephalus buckleyi* is 0.9 to 1.6%. Both species are sympatric at km 80 Pompeya-Iro road indicating the existence of reproductive barriers between them.

*Osteocephalus vilmae* differs from *Osteocephalus cannatellai* in having a larger tympanum (~1/4 of head length in *Osteocephalus vilmae* vs. ~1/5 in *Osteocephalus cannatellai*), and areolate flanks with big flattened warts (areolate with small flattened warts in *Osteocephalus cannatellai*). Mitochondrial DNA sequences show that *Osteocephalus vilmae* and *Osteocephalus cannatellai* are not sister species (Fig. 1). *Osteocephalus vilmae* differs from *Osteocephalus cabrerai* in (1) lacking prominent tubercles on the lower jaw, (2) having smooth outer edge of Finger IV (outer edge with fringe in *Osteocephalus cabrerai*), (3) having less webbing in the hands (in *Osteocephalus vilmae* webbing reaches two thirds of the distance between the ultimate and penultimate tubercle of Finger IV, in *Osteocephalus cabrerai* it reaches the proximal border of the ultimate tubercle; Figs 12 and 16), and (4) low to indistinct tubercles in the tarsus (prominent in *Osteocephalus cabrerai*).

A cream to bronze iris with black reticulations distinguishes *Osteocephalus vilmae* from *Osteocephalus deridens*, *Osteocephalus oophagus*, *Osteocephalus planiceps*, and *Osteocephalus taurinus* which have bronze to golden irises with black lines radiating from the pupil; iris coloration also differs from *Osteocephalus carri*, *Osteocephalus festae*, *Osteocephalus heyeri*, *Osteocephalus subtilis*, and *Osteocephalus verruciger* which have predominantly dark irises, and from *Osteocephalus leoniae* which have a bicolor iris (Jungfer 2010; Jungfer and Lehr 2001; Lynch 2002). *Osteocephalus vilmae* is larger than *Osteocephalus exophthalmus* (maximum male SVL in *Osteocephalus vilmae* 55.77 mm, *n =* 5; in *Osteocephalus exophthalmus* 32.7 mm, *n =* 3; Smith and Noonan 2001) and *Osteocephalus fuscifacies* (maximum SVL = 44.17, *n =* 21). Skin texture in the flanks distinguishes *Osteocephalus vilmae* (coarsely areolate) from *Osteocephalus mutabor* and *Osteocephalus yasuni* (smooth). *Osteocephalus inframaculatus* differs from *Osteocephalus vilmae* in coloration of the ventral surfaces of hindlimbs (bold brown blotches in *Osteocephalus inframaculatus* are absent in *Osteocephalus vilmae*; Jungfer 2010).

##### Description of holotype.

Adult male, 51.85 mm SVL, head length 18.9, head width 19.0, eye diameter 6.8, tympanum diameter 4.9, femur length 28.0, tibia length 28.7, foot length 22.1. Head narrower than body, nearly as wide as long; snout truncate in lateral and dorsal views; distance from nostril to eye longer than diameter of eye; canthus rostralis distinct and straight; loreal region concave; internarial area depressed; nostrils moderately protuberant, directed laterally; interorbital area flat, lateral margins of frontoparietals distinct through skin; eye large, strongly protuberant; tympanic membrane clearly evident, slightly wider than high, about two thirds of eye length, separated from eye by ca. 85% of its diameter; tympanic annulus distinct except dorsally where it is covered by supratympanic fold; posterior end of supratympanic fold reaches mid arm insertion. Arm slender, axillary membrane present, reaching one third of arm length; three small low tubercles present along ventrolateral edge of forearm; relative length of fingers I<II<IV<III; fingers bearing large, oval discs, that of third finger about three fourths of tympanum diameter; subarticular tubercles prominent, round to ovoid, bifid in distal subarticular tubercle of Finger IV; supernumerary tubercles present; palmar tubercle small, elongated; prepollical tubercle large, flat, elliptical; prepollex enlarged; large dark keratinous nuptial excrescences covering inner surface of prepollex almost reaching the proximal border of disk of thumb; webbing basal between fingers I and II; webbing formula of fingers I basal II1½—2½III2+—2IV. Medium sized to small tubercles on tibiotarsal articulation; scattered low tubercles on tarsus, more abundant on outer edge; small tubercles scattered along ventrolateral edge of foot; toes bearing discs slightly wider than long, smaller than those of fingers; relative length of toes I<II<V<III<IV; outer metatarsal tubercle ill defined, small, round; inner metatarsal tubercle large, ovoid; subarticular tubercles single, round, protuberant; supernumerary tubercles restricted to the soles; webbing formula of toes I1—2^–^II1—2III1—2IV2—1^–^V. Skin on dorsum, head, and dorsal surfaces of limbs shagreen, with scattered tubercles; minute keratinized conical tubercles present on the eyelids and dorsal surface of head; skin on flanks areolate with big flattened warts; skin on venter coarsely granular; skin on ventral surfaces of head and thighs granular, that on shanks smooth. Cloacal opening directed posteriorly at upper level of thighs; short simple cloacal sheath covering cloacal opening; round tubercles below vent; two distinct white tubercles ventrolateral to vent. Tongue cordiform, widely attached to floor of mouth; dentigerous processes of the vomers angular, adjacent medially, posteromedial to choanae, bearing 9 and 6 (left/right) vomerine teeth; choanae trapezoidal, oblique; vocal slits short and curved posteroventral to the angle of snout at the base of tongue; vocal sac barely distinct above the arm and below the ear.

*Color of holotype in preservative*. Dorsum brown with a single diffuse interorbital mark; dorsal surfaces of forearms brown with diffuse brown bands; dorsal surfaces of hindlimbs brown with diffuse dark brown marks on shanks and feet. Venter dirty cream with light brown spots, more abundant on posterior half of the body; ventral surfaces of hindlimbs and forelimbs dirty cream without marks but with distinct white tubercles on forearms; outer half of ventral surfaces of forearms dark brown; sides of head light brown with oblique white bar from posteroventral border of orbit to border of jaw, below tympanum; vertical diffuse brown bar below eye, anterior to white bar; area behind white bar and eye dark brown including periphery of tympanum; flanks dirty cream, areolate region with brown reticulation. Iris silver with a brown mid-horizontal line and thin black reticulations.

*Color of holotype in life*. Based on digital photograph (Fig. 15). Dorsum pale brown without marks; canthal region pale brown with diffuse pale green subocular mark and dark stripe along the posterior half of upper lip; tympanum pink; flanks light green without marks; dorsal surfaces of thighs and tarsus pale brown with greenish brown transversal bands, forearms greenish brown; tibia pale brown without marks; anterior and posterior surfaces of thighs, concealed surfaces of tibia, and metatarsus pale blue. Venter dirty cream with light brown spots, more abundant on posterior half of the body; ventral surfaces of hindlimbs and forelimbs dirty cream. Iris dirty cream with brown transversal midline and black reticulations.

##### Etymology.

The specific name is a patronym for Vilma Duran, in recognition of her continued work and efforts toward the improvement of the herpetological collection of CORBIDI and also for collecting the holotype and tissue of this new species.

##### Variation.

**Figure 17.**
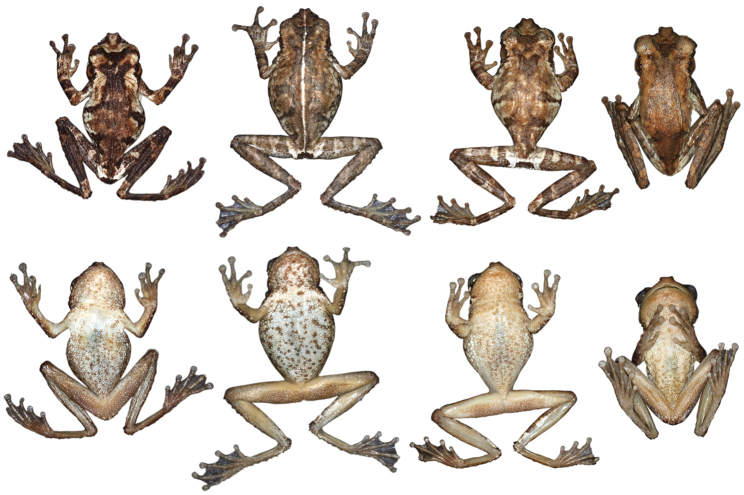
Adult male *Osteocephalus vilmae* showing variation in dorsal and ventral coloration of preserved specimens. Upper row, from left to right: CORBIDI 5031, CORBIDI 6469, CORBIDI 1086, CORBIDI 4773 (holotype), Peru, Región Loreto, Provincia Datem del Marañón, Jibarito, Capihuari Norte, Andoas, Pampa Hermosa.

Dorsal and ventral coloration of preserved specimens is shown in Figure 17. Dorsal background coloration varies from light brown to brown; irregular dark brown or dark gray marks are always present (Fig. 17). Flanks are always cream to grayish cream. Two specimens have a cream middorsal line from the tip of the snout to the vent (CORBIDI 06469, QCAZ 51205). The prominence of the tubercles can decrease in preserved specimens: when collected, CORBIDI 01086 had large conspicuous dorsal tubercle, in preservative tubercles are barely noticeable.

Ventral surfaces of preserved specimens (Fig. 17) vary from cream to vanilla. In most specimens, there are dark brown spots, more distinct posteriorly or in the throat (e.g., CORBIDI 06469); ventrally, limbs vary from dirty cream to light brown; all specimens have small white tubercles in the external edge of the forearm. The vent region is gray to brown with dark brown flecks or dots. Flanks are cream to gray, areolate, with dark brown reticulations, dots, and blotches along the entire flank or restricted to the posterior half (e.g. CORBIDI 05031).

Head shape is truncate in dorsal view and truncate in lateral view. Lateral head coloration varies from light brown with dark mottling (CORBIDI 01086) to grayish white with dark brown canthus rostralis and preocular stripes (CORBIDI 05031). All specimens have a white to cream subocular mark. The tympanic annulus is concealed dorsally and has lighter color than the background. The distal subarticular tubercle on Finger IV is bifid in all specimens.

Adult morphometric data are summarized in Table 3.In the examined series, the largest male has a SVL of 55.77 mm; mean male SVL = 50.74 mm (*n =* 6, SD = 3.17).

##### Color in life.

Based on a digital photograph of adult male CORBIDI 01086: dorsum light brown with irregular dark brown and light green marks; canthal region greenish brown with white subocular mark and dark brown band along posterior half of upper lip; tympanum pink contrasting with dark brown tympanic annulus; flanks light green with dark brown reticulation anteriorly and few irregular dark brown blotches posteriorly; dorsal surfaces of thighs, shanks and forelimbs brown with dark brown transversal bands; posterior surfaces of thighs light green; venter white speckled with light brown blotches; iris light cream with brown mid-horizontal line and fine black reticulations.

##### Distribution and ecology.

*Osteocephalus vilmae* is know from seven localities in the Peruvian and Ecuadorian Amazon basin (northern Loreto region), four at Río Corrientes (Jibarito, Nuevo Corrientes, Pampa Hermosa, and Shiviyacu), two near Rio Pastaza in the border Ecuador-Peru (Andoas and Capahuari Norte) and one at Provincia de Orellana, Pompeya-Iro road (Fig. 2). The elevations of these localities are between 150 to 270 m above sea level. Maximum airline distance between localities is 158 km. The Peruvian localities are dominated by *Terra Firme* forest. Specimens collected in Capahuari Norte were found in a stream surrounded by a mixture of primary and secondary forest. In Jibarito, Pampa Hermosa, and Shiviyacu the frogs were found in primary forest in a swamp close to a stream. All specimens were next to the streams, perching on tree branches 100 to 200 cm above the ground. *Osteocephalus vilmae* occurssympatrically with *Osteocephalus buckleyi* at km 80 Pompeya-Iro road. At the Peruvian localities it co-occurs with *Osteocephalus mutabor* and *Osteocephalus planiceps*.

**Table 3.** Descriptive statistics for morphometric measurements of species of the *Osteocephalus buckleyi *complex. Mean ± SD is given with range below. Bold figures are averages for individuals of all populations. Abbreviations are: SVL = snout-vent length; FOOT = foot length; HL = head length; HW = head width; ED = eye diameter; TD = tympanum diameter; TL = tibia length; FL = femur length. All measurements are in mm.

	**SVL**	**FOOT**	**HL**	**HW**	**ED**	**TD**	**TL**	**FL**
***Osteocephalus cabrerai***Males (*n = *7)	42.54 ± 2.51(39.66–45.72)	17.46 ± 0.92(16.15–18.57)	15.33 ±0.73(14.33–16.29)	14.86 ± 0.73(14.11–15.66)	4.11 ± 0.38(3.54–4.56)	3.4 ± 0.34(3–3.9)	23.62 ± 1.04(22.37–25.14)	21.93 ± 1.11(20.09–22.9)
***Osteocephalus cannatellai***Males (*n = *33)	**46.84 ± 4.31(38.46–57.21)**	**19.68 ± 2.05(15.96–24.30)**	**16.27 ± 1.48(13.86–19.10)**	**15.12 ± 1.94(11.39–19.80)**	**5.14 ± 1.65(4.24–6.40)**	**3.22 ± 0.48(2.16–4.21)**	**25.83 ± 2.47(20.68–31.45)**	**23.40 ± 2.52(18.87–29.0)**
Females (*n = *3)	66.55 ±5.44(62.64–72.77)	28.31 ± 2.69(26.12–31.32)	21.68 ± 1.25(20.76–23.11)	18.36 ± 1.59(17.3–20.2)	5.86 ± 0.43(5.42–6.28)	3.85 ± 0.16(3.7–4.02)	37.15 ± 3.25(34.17–40.62)	34.64 ±2.19(32.89–37.11)
BobonazaMales (*n = *2)	44.79 ± 2.72(42.86–46.72)	18.55 ± 0.80(17.98–19.12)	15.34 ± 1.39(14.36–16.33)	12.85 ± 0.45(12.53–13.17)	4.94 ± 0.12(4.85–5.03)	2.91 ± 0.18(2.78–3.04)	4.82 ± 1.64(23.66–25.98)	21.34 ± 0.69(20.85–21.83)
PomonaMales (*n = *3)	49.65 ± 5.54(43.67–54.61)	21.06 ± 2.69(18.5–23.88)	17.66 ± 1.72(15.27–18.31)	14.29 ± 1.41(12.66–15.23)	5.02 ± 0.68(4.44–5.78)	3.21 ± 0.36(2.83–3.56)	26.08 ± 2.98(23.52–29.36)	24.21 ±2.86(21.52–27.22)
YawiMales (*n = *5)	41.86 ± 2.91(38.46–45.46)	17.11 ± 0.78(15.96–18.1)	14.65 ± 0.95(13.86–16.24)	12.53 ± 0.98(11.39–13.67)	4.61 ± 0.42(4.24–5.34)	2.92 ± 0.47(2.16–3.48)	22.41 ± 1.21(20.68–23.77)	20.22 ± 1.05(18.87–21.69)
Female (*n = *1)	64.25	27.5	20.76	17.58	5.42	3.7	36.66	32.89
ZanjarajunoMales (*n = *2)	50.07 ± 0.96(49.39–50.75)	21.46 ± 1.34(20.51–22.41)	17.73 ± 0.07(17.68–17.79)	14.15 ± 0.16(14.03–14.27)	5.07 ± 0.09(5.01–5.14)	3.24 ± 0.16(3.13–3.36)	28.17 ± 2.24(26.59–29.76)	24.75 ± 1.36(23.79–25.75)
Female (*n = *1)	72.77	31.32	23.11	20.2	6.28	4.02	40.62	37.11
***Osteocephalus buckleyi***Males (*n = *14)	41.34 ± 2.41(38.01–45.25)	16.42 ± 1.07(14.51–18.34)	14.46 ± 0.74(13.05–15.82)	12.49 ± 1.26(10.84–15.35)	4.26 ± 0.30(3.76–4.84)	3.51 ± 0.19(3.20–3.88)	22.05 ± 1.21(20.07–24.24)	20.14 ± 1.20(17.76–22.37)
Females (*n = *2)	45.68 ± 7.44(40.42–50.95)	18.06 ± 1.52(16.99–19.14)	16.08 ± 2(14.67–17.5)	13.47 ± 1.49(12.42–14.53)	4.69 ± 0.53(4.32–5.07)	3.66 ± 0.2(3.52–3.81)	25.14 ± 3.63(22.57–27.71)	23.49 ± 2.8(21.51–25.47)
***Osteocephalus germani***Males (*n = *2 )	41.26–41.45	17.97–18.17	12.79–12.99	14.23–14.82	4.51–5.23	3.79–3.99	23.10–23.50	22.30–22.70
Females (*n = *2)	49.16–50.76	21.00–22.10	13.67–15.00	17.23–17.67	5.10–5.35	3.80–4.17	26.80–27.70	25.00–27.00
***Osteocephalus vilmae***Males (*n = *6)	50.74 ± 3.17(48.23–55.77)	21.06 ± 1.16(19.61–22.11)	16.78 ± 1.32(14.90–18.09)	18.03 ± 1.13(16.46–19.22)	6.092 ± 0.62(5.27–6.80)	4.43 ± 0.29(4.10–4.90)	27.90 ± 0.64(27.00–28.70)	25.93 ± 1.50(24.20–28.00)

**Table 4.** Descriptive statistics for call parameters of *Osteocephalus buckleyi* and *Osteocephalus cannatellai* sp. n. Mean ± SD is given with range below. The calls ofboth species have an obligatory first component consistent of a rattle-like note. *Osteocephalus cannatellai* has a facultative second component consistent of one to three quack notes. Sample sizes are number of males. Temporal characters are shown in seconds; spectral characters in Hertz.

	*Osteocephalus cannatellai*			*Osteocephalus buckleyi*
	Combined(n = 5)	Río Piraña(n = 1)	Río Pucayacu(n = 4)	Jatun Sacha(n = 2)
Duration of first component note	0.425 ± 0.053(0.356–0.489)	0.356	0.442 ± 0.042(0.389–0.489)	0.059 ± 0.004(0.056–0.063)
Call Rate	0.3066 ± 0.113(0.208–0.454)	0.454	0.269 ± 0.090(0.208–0.402)	1.524 ± 0.151(1.417–1.631)
First component interval	4.114 ± 1.722(2.142–6.004)	2.142	4.607 ± 1.528(2.568–6.004)	0.725 ± 0.140(0.625–0.824)
Dominant Frequency	1049.54 ± 247.18(771.6–1412.6)	771.616	1119.02 ± 221.99(765.68–1472.26)	745.66 ± 0.87(745.04–746.28)
Number of pulses	12.213 ± 1.585(9.8–14.2)	12	12.266 ± 1.825(9.8–14.2)	3.328 ± 0.181(3.2–3.457)
Pulse rate	28.932 ± 4.095(23.847–34.016)	33.661	27.749 ± 3.610(22.004–33.495)	55.833 ± 1.565(41.772–69.893)
Duration of second component	0.307 ± 0.106(0.216–0.488)	0.216	0.329 ± 0.108(0.25–0.488)	NA
Duration of second component note	0.032 ± 0.004(0.027–0.037)	0.027	0.033 ± 0.004(0.027–0.037)	NA
Number of second component notes	0.866 ± 0.339(0.445–1.287)	1	0.832 ± 0.381(0.225–1.439)	NA
Quack rate	0.140 ± 0.026(0.108–0.177)	0.108	0.148 ± 0.022(0.125–0.177)	NA

### Morphometric comparisons among species

**Figure 18.**
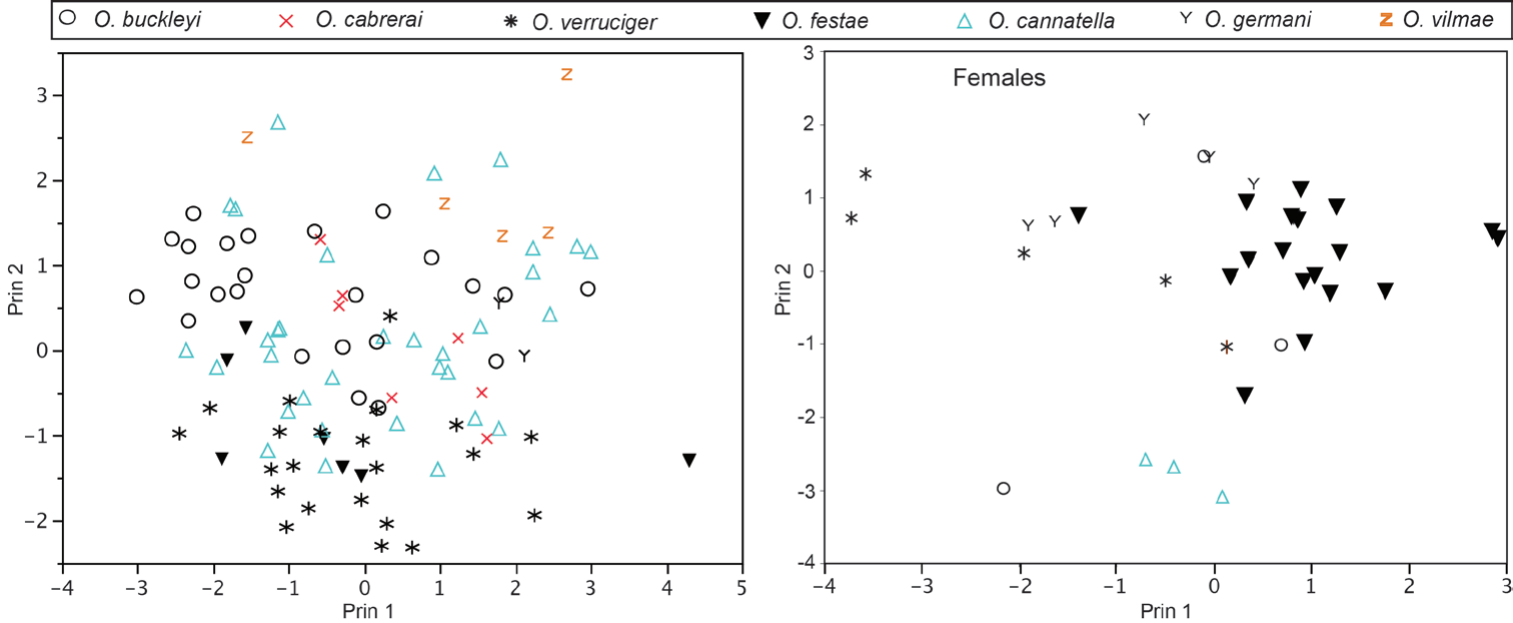
Principal components from analysis of seven size-corrected morphological variables. See Table 5 for character loadings on each component.

Three components with eigenvalues > 1.0, accounting for 70.9% of the total variation, were extracted from the PCA for males (Table 5). The highest loadings were femur length and tibia length for PC I, eye diameter and tympanum diameter for PC II, and head length for PC III (Table 5). Some species pairs have at least partly segregating morphometric spaces: *Osteocephalus festae*-*Osteocephalus buckleyi*, *Osteocephalus festae*-*Osteocephalus cabrerai*, *Osteocephalus vilmae*-*Osteocephalusbuckleyi*, and *Osteocephalus vilmae*-*Osteocephalus cabrerai* (Fig. 18).* Osteocephalus germani* does not overlap with *Osteocephalus vilmae*, *Osteocephalus festae* and *Osteocephalus cabrerai* but this differentiation requires to be verified with larger sample sizes for *Osteocephalus germani* (currently *n* = 2). Principal Component I mainly describes hindlimb length (Table 5). There is low interspecies differentiation along PC I. Species with low scores on PC II are *Osteocephalus festae* and *Osteocephalus verruciger*; *Osteocephalus vilmae* has high scores. Pairwise comparisons between *Osteocephalus festae* and all other species (except *Osteocephalus verruciger*) are significant (all *P* values for *t* tests < 0.04); *Osteocephalus verruciger* also shows significant differences with all the remaining species (all *P* values < 0.004).

Three components with eigenvalues > 1.0 were extracted from the PCA for females (Table 5). The three components accounted for 75.7% of the total variation. The highest loadings for the PCA for females were tibia length and femur length for PC I, head width for PC II, and eye diameter and head length for PC III (Table 5). As in the PCA for males, there is wide overlap in morphometric space among species (Fig. 18). The only exception is *Osteocephalus cannatellai*, which segregates from the other species along PC II . However, larger sample sizes are required to confirm this differentiation.

In the DFA classification on males, all *Osteocephalus cabrerai*, *Osteocephalus festae*, *Osteocephalus germani*, *Osteocephalus verruciger*, and *Osteocephalus vilmae* were correctly classified (*n* = 7, 7, 2, 22, and 5 respectively). In *Osteocephalus buckleyi*, 17 out of 24 specimens were correctly classified (4 were misclassified as *Osteocephalus cabrerai*, 2 as *Osteocephalus germani*, and 1 as *Osteocephalus vilmae*); in *Osteocephalus cannatellai*, only 4 out of 33 specimens were incorrectly classified, 2 as *Osteocephalus buckleyi* and 2 as *Osteocephalus vilmae*. Overall, the DFA show morphometric differentiation among the analyzed species. The DFA on females shows even better discrimination because all individuals were correctly assigned to their own species.

**Table 5.** Character loadings and eigenvalues for Principal Components (PC) I–III. The analysis was based on seven morphometric variables of adult *Osteocephalus buckleyi*, *Osteocephalus cabrerai*, *Osteocephalus cannatellai* sp. n., *Osteocephalus festae*, *Osteocephalus germani* sp. n., *Osteocephalus verruciger* and *Osteocephalus vilmae* sp. n. Bold figures indicate highest loadings.

**Variable**	**PCA Males**			**PCA Females**		
	**PC I**	**PC II**	**PC III**	**PC I**	**PC II**	**PC III**
Femur length	**0.557**	0.126	0.052	**0.553**	0.030	0.193
Foot length	0.425	–0.400	0.046	0.451	0.374	–0.106
Head length	0.106	0.459	**0.715**	–0.004	–0.326	**0.649**
Head width	0.433	–0.053	–0.480	0.161	**0.652**	–0.083
Eye diameter	0.173	**0.554**	–0.332	–0.018	0.349	**0.690**
Tympanum diameter	0.066	**0.540**	–0.233	–0.376	0.421	0.201
Tibia length	**0.525**	–0.108	0.298	**0.568**	–0.164	0.080
Eigenvalue	2.451	1.432	1.083	2.311	1.768	1.222

## Discussion

Similarly to previous studies on Amazonian amphibians (e.g., Elmer et al. 2007; Fouquet et al. 2007; Funk et al. 2011) our results document a large proportion (300% increase) of hidden diversity within a set of populations that were previously treated as a single widely distributed species. Moreover, because most of our sampling was restricted to Ecuador and Peru, it is likely that there are even more species than found in our study. These results highlight the need to carry out large-scale genetic surveys of Amazonian amphibians to achieve a more realistic understanding of their diversity and evolution.

Genetic evidence is a valuable taxonomic tool but, in most cases, is insufficient to define species boundaries without reference to other sets of characters like advertisement calls or external morphology. Taxonomic reviews of Amazonian amphibians suggest that morphological characters are too conservative to define species boundaries because closely related species share similar morphology (e.g., Elmer et al. 2007; Fouquet et al. 2012; Funk et al. 2011; Lougheed et al. 2006; Padial et al. 2009). Our results, however, indicate that in some groups, like the *Osteocephalus buckleyi* species complex, this is not necessarily the case. The three new species described here are diagnosable with morphological evidence alone and are distinctive from the other species of the complex. Morphological differences are also evident between *Osteocephalus buckleyi*, *Osteocephalus cabrerai*, *Osteocephalus carri*, *Osteocephalus festae*, and *Osteocephalus mutabor*. Thus, none of the species of the complex are strictly cryptic (i.e., all of them can be identified using morphological characters) although their diagnosis based on morphology is challenging. Other groups of Amazonian amphibians on which phylogenetic analyses of DNA have led to the discovery of species that turned out to be morphologically distinct are the *Hypsiboas fasciatus-calcaratus* complex (Funk et al. 2011) and the *Pristimantis* “*ockendeni*” complex (Elmer and Cannatella 2008).

We suspect that the difficulty in defining species boundaries based on morphology arises from the high intraspecific polymorphism in coloration characteristic of most groups of dull-colored Amazonian amphibians like *Osteocephalus* and *Pristimantis* (see for example Fig. 2 in Elmer and Cannatella 2008 and Figs 3, 8, 14, 17 herein). If this is the case, understanding the evolutionary processes that generate and maintain polymorphism in coloration could help to predict which Amazonian taxa are more likely to contain “cryptic” diversity. One plausible process is frequency dependent predation which, occurs when the probability of predation is inversely correlated to the frequency of a given prey type in the population (for a review see Punzalan et al. 2005). Under this scenario, predators use search images to find preys and are better at detecting previously seen prey types because they have learned to find them. Although other processes could also explain polymorphisms (e.g., deferential selection associated with spatial variation in backgrounds), the available evidence suggests that some form of frequency dependent selection is the most likely explanation for color polymorphism in anurans (Milstead et al. 1974; Wells 2007, pp. 715).

Most *Osteocephalus* have a predominantly and highly polymorphic brown coloration and are cryptic against the background where they are found by day (Deichmann 2008; Deichmann and Williamson 2007; SRR pers. obs.) If polymorphisms are an adaptation to avoid falling into search categories of visually oriented predators, the difficulties of species delimitation based on morphological characters could be a byproduct of this selective pressure. This hypothesis needs to be tested empirically because if verified it could help to understand why several groups of Neotropical amphibians contain a large proportion of cryptic species.

### Biogeography and speciation

Examination of the geographic ranges of sister species can provide insights into modes of speciation. Our phylogeny of the *Osteocephalus buckleyi* species complex recovered four sister species pairs of which one is sympatric (*Osteocephalus buckleyi*-*Osteocephalus vilmae*) and three are allopatric. Among the allopatric pairs, two involve a lowland species sister to a highland species. *Osteocephalus mutabor* occurs at lower altitudes (range 230–1240 m) than its sister species, *Osteocephalus festae* (860–2383 m). Similarly, *Osteocephalus cannatellai* has a lower distribution (200–1290 m) than its sister species, *Osteocephalus verruciger* (950–2120 m). Because most species of *Osteocephalus* are restricted to elevations below 1000 m, the distributions of *Osteocephalus festae* and *Osteocephalus verruciger* probably represent parallel and recent colonization events from the lowlands. This geographic pattern suggests that speciation has been a result of ecological mediated selection along an altitudinal gradient. Interestingly, both highland species resemble each other closely in external morphology (Figs 15 and 18) suggesting convergence as a byproduct of adaptation to similar environments. Speciation associated with ecological divergence along altitudinal gradients was also reported by Graham et al. (2004) in dendrobatid frogs and more recently by Salerno et al. (2012) between *Tepuihyla* and its sister lowland species, “*Osteocephalus*” *exophthalmus*.

At the intraspecific level, we found low genetic divergence with the only exception of *Osteocephalus festae* (up to 2.8% of uncorrected *p* distance in gene 12S). We also found a concordant geographic pattern of divergence in *Osteocephalus buckleyi* and *Osteocephalus mutabor* because in both the most divergent population was the most northern of them, in the Cuyabeno region. High divergence of samples from Cuyabeno relative to others to the south was also reported for *Pristimantis kichwarum* (Elmer and Cannatella 2008). Samples of *Osteocephalus mutabor*, *Osteocephalus buckleyi* (*sensu stricto*) and *Osteocephalus cannatellai* show genetic structure generally congruent with geography (i.e., geographically close localities tend to be genetically similar). Overall, our intraspecific sampling reveals low levels of genetic differentiation and genetic variation geographically structured.

## Supplementary Material

XML Treatment for Osteocephalus cannatellai

XML Treatment for Osteocephalus germani

XML Treatment for Osteocephalus vilmae

## Appendix I

*Osteocephalus alboguttatus*. ECUADOR: PROVINCIA PASTAZA: Canelos (QCAZ 15981).

*Osteocephalus buckleyi*. ECUADOR: PROVINCIA NAPO: Cando, 700 m (QCAZ 24446–48); Cabañas Pimpilalo, 600 m (QCAZ 23043); Ahuano, 410 m (QCAZ 36703); Jatun Sacha, 420 m (QCAZ 2876, 48093, 48827); Serena, Río Jatunyacu, 520 m (QCAZ 25321); Tena, 550 m (QCAZ 8809–10); Juan Pablo II, Río Punino, 360 m (EPN-H 5476); Santa Rosa de Arapino, 570 m (EPN-H 6209); Nuevo Rocafuerte, Bloque 3, Pozo PCSA 1, 210 m (EPN-H 6516); PROVINCIA ORELLANA: El Edén, 235 m (QCAZ 33148); Río Yasuní (QCAZ 7360); Puente del Río Beque, 228 m (QCAZ 43071); Río Rumiyacu, Parque Nacional Yasuní, 250 m (QCAZ 16007); Taracoa, 251 m (QCAZ 34963); Aguarico, Parque Nacional Yasuní , Pozo Ewa, 320 m (EPN-H 2785–87); Pozo Sunka, 279 m (EPN-H 2757–58); Dayuano, Cotapino, 500 m (EPN-H 7471); Guiyero, 248 m (EPN-H 10909); Comuna Huatarco, Pozo Chonta, 300 m (EPN-H 11710, 11717–18); PROVINCIA PASTAZA: Canelos, 650 m, (BMNH 1947.2.13.44; lectotype); Tarangaro, 338 m (QCAZ 39073–74, 39081–82, 39146, 39172, 39191); Villano, 380 m (QCAZ 38503, 38704–05, 38713, 39285); Kurintza, 380 m (QCAZ 39033); PROVINCIA SUCUMBÍOS: Tarapoa (QCAZ 14948); Puerto Bolívar, 240 m (QCAZ 28231); Playas de Cuyabeno, 230 m (QCAZ 28277, 28280, 28395); Tarapoa-Puerto Carmen road, bridge over Río Cuyabeno, 290 m (QCAZ 28427); Comuna Shuar Chari, 280 m (EPN-H 4875); Cascales, Pozo Diamante, 410 m (EPN-H 5418, 5475); La Barquilla (EPN-H 7524–26); PERU: REGION LORETO: Provincia de Requena: Sierra del Divisor, 500 m (CORBIDI 03747); Provincia Datem del Marañón: Singasapa, 186 m (CORBIDI 06522); Sargento Puño, 206 m (CORBIDI 07458–59, 07462, 07473, 07516, 07558, 07560, 08675, 08714); PROVINCIA DE MAYNAS: Río Momon-San Luis de Vista Alegre (Puchana), 108 m (CORBIDI 2980–81).

*Osteocephalus cabrerai*. ECUADOR: PROVINCIA SUCUMBÍOS: Cuyabeno, Campamento Concienti, 260 m (EPN-H 7201–5); Puerto Bolívar, 240 m (QCAZ 27923, 28231); Campamento Guepicillo, 220 m (CORBIDI 00119–22, 00200); PERU: REGION LORETO: Provincia de Requena: Sierra del Divisor, 500 m (CORBIDI 02452, 02632, 05064); Provincia de Maynas: Gueppi, 220 m (CORBIDI 00199); Rio Yanayacu, Campamento Curupa, 125 m (CORBIDI 05819–21, 05831).

*Osteocephalus deridens*. ECUADOR: PROVINCIA ORELLANA: Estación Científica Yasuní, Universidad Católica del Ecuador, Parque Nacional Yasuní (QCAZ 12556).

*Osteocephalus festae*. ECUADOR: PROVINCIA LOJA: San Francisco, Arco Iris Reserve, Parque Nacional Podocarpus (3.9884°S, 79.0930°W), 2200 m (QCAZ 39364); PROVINCIA MORONA SANTIAGO: Río Napinaza, 6.6 km N from General Leonidas Plaza (Limón) in the road to Mendez (2.9266°S, 78.4070°W), 1010 m (QCAZ 26283, 26304, 26488, 26552, 26561, 32835, 38081, 38420, 39799, 39804–6, 39798–803, 39808–12); San Carlos, San Miguel and Río Oro, 600–1200 m (QCAZ 11624–26); PROVINCIA ZAMORA CHINCHIPE: Miasí Alto (4.2502°S, 78.6174°W), 1250–1300 m (QCAZ 41039); Reserva Tapichalaca (4.5500°S, 79.1291°W), 1637 m (QCAZ 45674); PERU: REGIÓN DE AMAZONAS: PROVINCIA DE BAGUA: Cataratas de Paraiso-Chonza Alta (5.6026°S, 78.398°W), 1342 m (CORBIDI 760–64, 758–59); Camñopite (5.6147°S, 78.3319°W), 1650 m (CORBIDI 1962–65, 2992); PROVINCIA DE BONGARA: Quebrada Goca (on Yambrasbamba road) (5.7641°S, 77.9129°W), 1711 m (CORBIDI 10461); PROVINCIA DE YAMBRASBAMBA: Quebrada Goca (on Yambrasbamba road) (5.7641°S, 77.9129°W), 1711 m (CORBIDI 10461); REGIÓN DE SAN MARTÍN: PROVINCIA MARISCAL CACERES: Río Lejia (6.8365°S, 77.4860°W), 1500 m (CORBIDI 623, 624); PROVINCIA RIOJA: Bajo Naranjillo (5.8157°, 77.3367°W), 844 m (CORBIDI 3386) ); PROVINCIA LAMAS: Cataratas de Ahuashiyacu (6.4174°S, 76.2893°W), 600 m (CORBIDI 09585, 09587).

*Osteocephalus fuscifacies*. ECUADOR: PROVINCIA NAPO: El Tena-Talag Road, 15 km from Tena, 550 m (QCAZ 8806); PROVINCIA ORELLANA: Pompeya-Iro Road, 38 km SE from Pompeya (QCAZ 8137); Estación Científica Yasuní, Universidad Católica del Ecuador, 240 m (QCAZ 20785).

*Osteocephalus leoniae*. —PERU: Región CUSCO: Provincia La Convención: Tangoshiari, 1135 m (CORBIDI 00306); Región SAN MARTIN: Provincia de Rioja: El Dorado, 844 m (CORBIDI 01415).

*Osteocephalus mutabor*. ECUADOR: PROVINCIA MORONA SANTIAGO: Cantón Morona, Nuevo Israel, 1290 m (QCAZ 46470–71); PROVINCIA NAPO: Chontapuntas, Comunidad Sumak Sacha-Pozo Yuralpa Centro 1 (QCAZ 28646–48); Huino, around the waterfall (QCAZ 30916–17, 30919–20, 30922–23, 30925–26); PROVINCIA ORELLANA: km 22 Pompeya-Iro Road, 287 m (QCAZ 42999); PROVINCIA PASTAZA: Pomona, Fundación Hola Vida, 846 m (QCAZ 25603, 25684); Cantón Santa Clara, Río Pucayacu, Colonia Mariscal Sucre (QCAZ 29430, 36935, 36946, 40253); Canelos (QCAZ 41030); PROVINCIA SUCUMBIOS: Puerto Bolívar, 240 m (QCAZ 28223). PERU: REGION LORETO, Provicia Loreto: Andoas, 187 m, (CORBIDI 04645); REGION AMAZONAS: Cordillera de Kampankis (CORBIDI 09369).

*Osteocephalus planiceps*. ECUADOR: PROVINCIA DE NAPO: Chontapuntas, Comunidad Sumak Sacha-Pozo Yuralpa Centro 1 (QCAZ 28648); PROVINCIA ORELLANA: Parque Nacional Yasuní, km 38 Pompeya-Iro Road, 280 m (QCAZ 5134, 14842); Estación Científica Yasuní, Universidad Católica del Ecuador, 240 m (QCAZ 14844, 20797–800); PROVINCIA SUCUMBIOS: La Selva lodge, 250 m (QCAZ 7408, 12093–95).

*Osteocephalus taurinus*. ECUADOR: PROVINCIA ORELLANA: Parque Nacional Yasuní, km 97 Pompeya-Iro road, 450 m (QCAZ 5301); Estación Científica Yasuní, Universidad Católica del Ecuador, 220 m (QCAZ 9007, 10604, 14804, 14954, 24449–50); PROVINCIA SUCUMBÍOS: Reserva de Producción Faunística Cuyabeno, 220 m (QCAZ 5871–77); Puerto Bolívar, 240 m (QCAZ 27916, 27920); Zábalo, 220 m (QCAZ 27982, 28015); Chiritza-Puerto El Carmen road, bridge over Río Aguas Negras, 270 m (QCAZ 28485); Tarapoa-Puerto El Carmen road, bridge over Río Cuyabeno, 290 m (QCAZ 28435–36); PROVINCIA ZAMORA CHINCHIPE: Shaime, Nangaritza, 980 m (QCAZ 18230).

*Osteocephalus verruciger*. ECUADOR: PROVINCIA MORONA SANTIAGO: Nueve de Octubre (QCAZ 32266); Bosque Protector Abanico, Morona, 1647 m (EPN-H 11444); Morona, 1530 m (EPN-H 11445); Río Sardinayacu, Palora, Parque Nacional Sangay, 1600 m (EPN-H 5940–42, 5947); PROVINCIA NAPO: near Santa Rosa de Quijos, 1661 m (QCAZ 45344); E of Volcán Sumaco, 1570 m (QCAZ 1560, 1562); Nueva Loja road between Cascabel 1 and 2, 1600 m (QCAZ 7783–84); Río Salado (QCAZ 17285); Sumaco, 1800–2100 m (QCAZ 8964); Pacto Sumaco (QCAZ 10907); km 13 Loreto-Coca road, 1324 m (QCAZ 22201); Río Hollín (QCAZ 1681, 2405); Coordillera de los Guacamayos, Cosanga-Archidona road, 1600 m (QCAZ 12206, 41108); El Reventador (QCAZ 29208); Cascada San Rafael, 1553 m (QCAZ 363, 13225,13247, 16954, 32032–36); Cosanga, 339 m (QCAZ 15942); PROVINCIA SUCUMBIOS: Quito-Lago Agrio road, Río Azuela, 1680 m (QCAZ 15149, 15991–97, 16220, 16953, 22497, EPN-H 6341, 11987, 12105–07, 12112, 12143); La Bonita (QCAZ 3175); Rosa Florida, 1185 m (QCAZ 20544); trail to Volcán Reventador, Gonzalo Pizarro, 1800 m (EPN-H 7052–53, 7059–60); PERU: REGIÓN DE AMAZONAS: Provincia Condorcanqui: Cabecera de la Quebrada Katerpiza, 1100 m (CORBIDI 09477); REGIÓN DE LORETO: Provincia Datem del Marañón: Cabecera de la Quebrada Wee, 1000 m (CORBIDI 09525).

*Osteocephalus yasuni*. ECUADOR: PROVINCIA SUCUMBIOS: Zábalo, 220 m (QCAZ 27998); Playas de Cuyabeno, 230 m (QCAZ 27816).

*Trachycephalus jordani*. ECUADOR: PROVINCIA EL ORO: Bosque Protector Puyango (QCAZ 35405).

*Trachycephalus typhonius*. ECUADOR: PROVINCIA PASTAZA: km 6 San Ramón-El Triunfo road, Colonia Mariscal Sucre, trail to Río Pucayaku (QCAZ 38075).
